# Metabarcoding analysis of the stomach contents of the Antarctic Toothfish (*Dissostichus mawsoni*) collected in the Antarctic Ocean

**DOI:** 10.7717/peerj.3977

**Published:** 2017-11-07

**Authors:** Tae-Ho Yoon, Hye-Eun Kang, Soo Rin Lee, Jae-Bong Lee, Gun Wook Baeck, Hyun Park, Hyun-Woo Kim

**Affiliations:** 1 Interdisciplinary Program of Biomedical, Mechanical and Electrical Engineering, Pukyong National University, Busan, Republic of Korea; 2 Department of Marine Biology, Pukyong National University, Busan, Republic of Korea; 3 National Institute of Fisheries Science (NIFS), Busan, Republic of Korea; 4 Department of Seafood & Aquaculture Science/Institute of Marine Industry/Marine Bio-Education & Research Center, College of Marine Science, Gyeongsang National University, Tongyeong, Republic of Korea; 5 Korea Polar Research Institute, Korea Ocean Research and Development Institute, Incheon, Republic of Korea

**Keywords:** Metabarcoding, Antarctic toothfish, Foodweb, Stomach contents, Antarctica, Next-generation sequencing

## Abstract

Stomach contents of the Antarctic toothfish, *Dissostichus mawsoni*, collected from subareas 58.4 and 88.3, were analyzed using next generation sequencing (NGS) technology. After processing the raw reads generated by the MiSeq platform, a total of 131,233 contigs (130 operational taxonomic units [OTUs]) were obtained from 163 individuals in subarea 58.4, and 75,961 contigs (105 OTUs) from 164 fish in subarea 88.3. At 98% sequence identity, species names were assigned to most OTUs in this study, indicating the quality of the DNA barcode database for the Antarctic Ocean was sufficient for molecular analysis, especially for fish species. A total of 19 species was identified from the stomach of *D. mawsoni* in this study, which included 14 fish species and five mollusks. More than 90% of contigs belonged to fish species, supporting the postulate that the major prey of *D. mawsoni* are fish. Two fish species, *Macrourus whitsoni* and *Chionobathyscus dewitti*, were the most important prey items (a finding similar to that of previous studies). We also obtained genotypes of prey items by NGS analysis, identifying an additional 17 representative haplotypes in this study. Comparison with three previous morphological studies and the NGS-based molecular identification in this study extended our knowledge regarding the prey of *D. mawsoni*, which previously was not possible. These results suggested that NGS-based diet studies are possible, if several current technical limitations, including the quality of the barcode database or the development of precise molecular quantification techniques to link them with morphological values, are overcome. To achieve this, additional studies should be conducted on various marine organisms.

## Introduction

The Antarctic toothfish, *Dissostichus mawsoni*, belongs to the family Nototheniidae of the order Perciformes, which is native to the Southern Ocean. It is generally distributed in waters with subzero temperatures below the latitude of 60°S. *Dissostichus mawsoni* is an economically important fishery resource and its commercial catch in 2015 was 624 tons in subarea 88.2, according to the 2015 fishery report of the Commission for the Conservation of Antarctic Marine Living Resources (CCAMLR) (https://www.ccamlr.org/en). This species is also considered one of the most important components of the Antarctic pelagic food web together with *Pleuragramma antarcticum* (Eastman, 1993). Although it is a top fish predator with a voracious appetite, *D. mawsoni* is a source of food for cetaceans and seals (Calhaem & Christoffel, 1969; Yukhov, 1971). Its feeding ecology has been examined in different sampling sites, including the McMurdo Sound (Calhaem & Christoffel, 1969), the Pacific sector of Antarctica (Yukhov, 1971), Ross Sea (Fenaughty, Stevens & Hanchet, 2003; Stevens et al., 2014), the South Sandwich Islands (Roberts, Xavier & Agnew, 2011), and the Lazarev Sea (Petrov & Tatarnikov, 2011). All the diet studies on *D. mawsoni* have been completed using direct observation of prey items, which is primarily dependent on the morphological characteristics of each prey species. Although most of the prey mass (90%) generally consisted of finfish and cephalopods, specific species differed among studies.

Although it has been successfully used in diet studies, morphological analysis has potential limitations when used alone. First, many prey items lose their morphological characteristics during the ingestion or digestion process, which makes their morphological classification difficult (Hashimoto, 1974). In addition, long-term training is necessary to obtain the professional knowledge needed to identify partially digested prey items. Second, digestion time differs for each prey item and morphological analysis may be biased toward recently consumed prey (Mata-Sotres et al., 2016). It is more difficult to quantitatively analyze the prey organisms that are present in a liquid state after being mixed with digestive juices. Third, morphological analysis takes extensive time and effort, which can be a bottleneck in the completion of large scale analyses.

Molecular identification of prey items is now being used to complement morphological analysis (Carreon-Martinez et al., 2011; Paquin et al., 2014). Because of the large number of DNA sequences in databases, including GenBank (http://www.ncbi.nlm.nih.gov/genbank) and the Barcode of Life Database (BOLD; http://www.boldsystems.org) (Ratnasingham & Hebert, 2007), there is a high likelihood of accurate species identification. Recently developed Next Generation Sequencing (NGS) technology enables researchers to obtain large quantities of DNA sequence data at a relatively low cost. In fact, fish stomach contents have already been analyzed using NGS technology with relatively low cost and accurate results (Harms-Tuohy, Schizas & Appeldoorn, 2016; Pompanon et al., 2012). In the present study, we used NGS to analyze the stomach contents of the Antarctic fish, *Dissostichus mawsoni*, and we compared them with data obtained from morphological analysis. For the NGS analysis, we used the Illumina MiSeq platform, which is the most economical choice with a low sequencing error rate (Luo et al., 2012; Quail et al., 2012). The DNA markers were in the cytochrome c oxidase I (COI) region, and were designed to cover most of the metazoan taxa and to be a suitable size for the MiSeq platform.

## Materials and Methods

### Collection and preparation of samples

**Figure 1:**
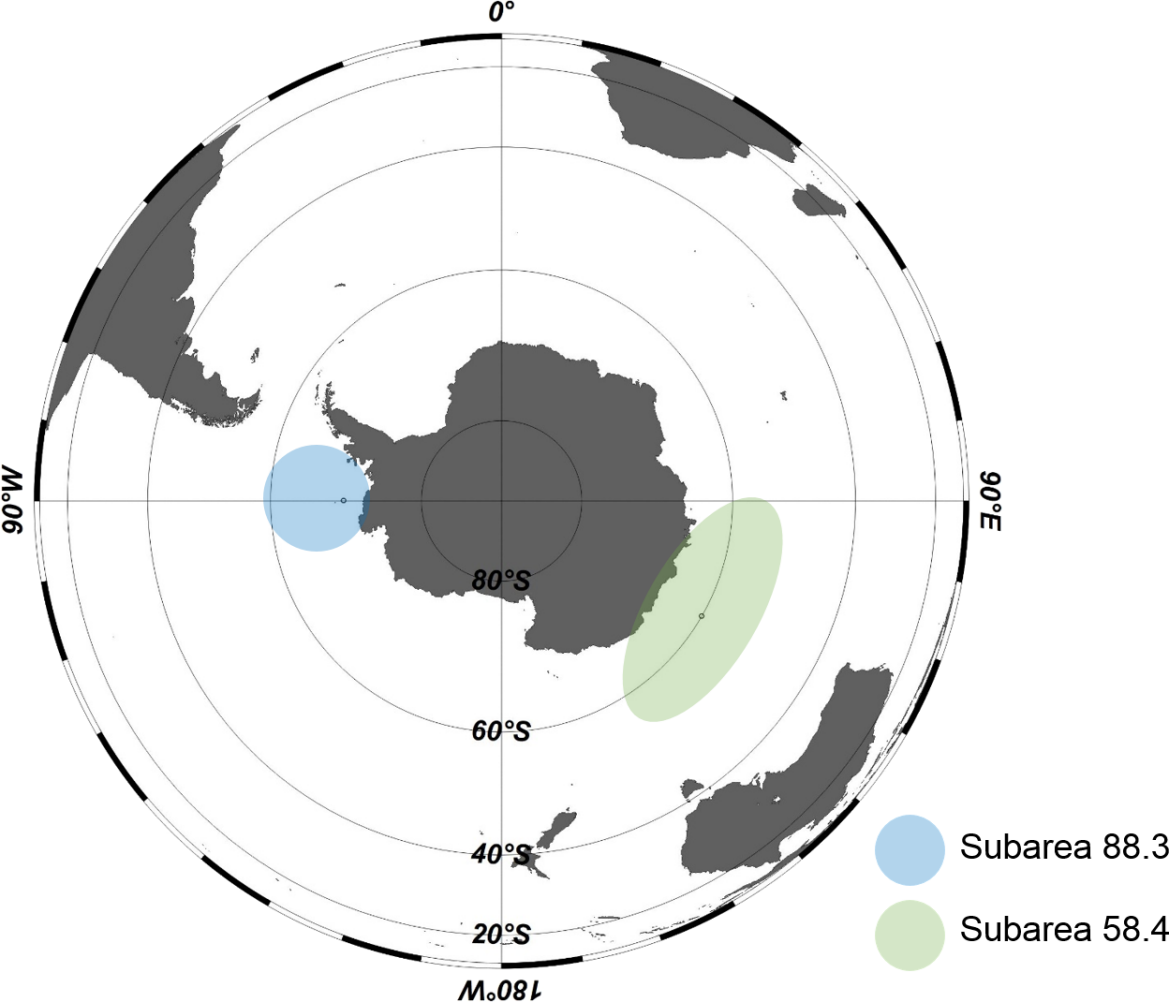
Areas in Antarctica for Antarctic toothfish (*Dissostichus mawsoni*) samples. Samples (*Dissostichus mawsoni*) were collected in subarea 58.4 and 88.3, research blocks in the Commision for the Conservation of Antarctic Marine Living Resources (CCAMLR) convention area from December 2015 to March 2016.

The samples used in this study were collected from Antarctic waters by a bottom longline from December 2015 to March 2016. Sample sites were in subarea 58.4 and 88.3, research blocks in the Commission for the Conservation of Antarctic Marine Living Resources (CCAMLR) convention areas (http://www.ccamlr.org/node/86816) (Fig. 1). Humboldt squid (*Dosidicus gigas*) and Pacific herring (*Clupea pallasii*) were used as baits. The depth of the fishing area was from 935 to 1,515 m in subarea 58.4 and from 871 to 1,628 m in subarea 88.3. Body size (cm) and weight (g) of each individual were measured at the collection sites, and the stomach was dissected and immediately frozen at −30 °C. The frozen stomachs of *Dissostichus mawsoni* were transported to the laboratory from the ship.

### Purification of genomic DNA and next generation sequencing

Frozen stomach contents were isolated from the stomach after thawing the fish at 4 °C. Wet weight of the contents of each stomach was measured and an equal amount of deionized water was added. The mixture was then completely homogenized using an electric kitchen blender and stored at −20 °C before DNA purification. To prevent cross-contamination, blenders were cleaned with bleach before use and only one blender was used for the samples from each subarea. Based on its proportion relative to the total weight of all stomach contents, homogenized contents of each stomach were pooled together into two groups; subarea 58.4 and subarea 88.3. Genomic DNA was extracted from the two pooled samples using the AccuPrep^®^ genomic DNA extraction kit (Bioneer, Daejeon, Republic of Korea) following the manufacturer’s instructions. Each pooled DNA sample was mixed with six volumes of lysis buffer and homogenized again using the Tissuelyser II (Qiagen, Hilden, Germany). Isolated genomic DNA was quantified and qualified by the Nano Drop Spectrophotometer ND-1000 (Thermo Scientific, Waltham, MA, USA).

To construct the library for the MiSeq platform, universal PCR primer sets were designed by comparing 15,564 sequences from the COI region, which covered 25 metazoan phyla (Datas S1 and S2). To reduce the bias produced by the differing efficiency of degenerated universal primer sequences, a nested PCR strategy was adopted with low cycle numbers. The first PCR was performed with two primers (COIMISQF1; 5′-ATNGGNGGNTTYGGNAA-3′ and COIMISQR1; 5′-TANACYTCNGGRTGNCC-3′). The expected amplified size for the first PCR was between 500 and 509 bp. The first PCR reaction mixture (20 µL) contained 100 ng of template, 1 μL of each primer (100 pmol), 2 µL of dNTPs (10 mM), 0.2 µL of Phusion High Fidelity DNA polymerase (New England Biolabs, Hitchen, UK), and 4 μL of 5× buffer. The first amplicons were purified using AccuPrep^®^ PCR Purification Kit (Bioneer, Daejeon, Republic of Korea) and eluted with 20 µL of elution buffer. The amplification reaction was performed using the following cycling conditions: initial denaturation at 94 °C for 3 min, followed by 15 cycles of 94 °C for 30 s, 48 °C for 30 s, and 72 °C for 30 s, with a final extension of 72 °C for 3 min. The PCR reaction without template DNA was performed as the negative control. The second PCR was performed to apply the Nextera XT index kit (Illumina, Hayward, CA, USA), as well as increase the specificity of obtained COI sequences. A forward PCR primer (NXCOIMISQF2; 5′-TCGTCGGCAGCGTCAGATGTGTATAAGAGACAGGGNGGNTTYGGNAAYTG-3′) and a reverse primer (NXCOIMISQF2; 5′-GTCTCGTGGGCTCGGAGATGTGTATAAGA GACAGGGRTGNCCRAARAAYCA-3′) were designed. To obtain sufficient products from the first PCR to attach adapter sequences, three different first PCR reactants were pooled and used as the template for the second PCR. The second PCR reaction mixture was the same as that of the first PCR, except for the addition of 4 µL of purified first PCR products and 1 µL of second PCR primers (100 pmol). Libraries were constructed from the purified second PCR amplicons using the Nextera XT index kit (Illumina, Hayward, CA, USA) according to the manufacturer’s instructions. Constructed libraries were identified by 1.5% agarose gel electrophoresis (approx. 630 bp) and further purified using the AccuPrep^®^ Gel Purification Kit (Bioneer, Daejeon, Republic of Korea). The constructed library was quantified by the qubit dsDNA HS Assay Kit (Invitrogen, Carlsbad, CA, USA) and its integrity was determined using the 2100 Bioanalyzer (Agilent Technologies, Santa Clara, CA, USA). Sequencing was performed using the Illumina MiSeq (2 * 300 bp paired-ends).

### Bioinformatic analysis of sequence data

After adapter/index sequences were trimmed from the obtained raw reads, reads with low quality (QV < 20) and short-read length (fewer than 100 nucleotides) were discarded using CLC Genomics Workbench v8.0 (CLC Bio, USA). The paired-end contigs were constructed with the Mothur software package v 1.35.0 (Schloss et al., 2009) using a cut-off of 480–510 bp in length, more than 6 bp in the overlapping sequences, and no mismatch. After primer sequences were trimmed, the obtained paired-end contigs were clustered at 99.6% identity and assigned operational taxonomic units (OTUs) using UCHIME software (Edgar et al., 2011). Finally, chimeric sequences were eliminated based on the *de novo* chimera detection. After the OTUs with low numbers (<2 OTUs) were removed, a BLASTn search was used against the NCBI non-redundant nucleotide database (*e*-value < 1e−50). Species names were assigned for OTUs with more than 98% sequence identity, and genus names were assigned for those with identities between 90% and 98%. OTUs with less than 90% sequence identity were described as “Unknown.” The OTUs assigned as *D. mawsoni* were removed from further analysis. The OTUs with less than 10 contig numbers were excluded from the analysis to remove possible artefacts that can occur during Miseq running (Yamamoto et al., 2017). A phylogenetic tree of representative haplotypes was constructed using the Minimum-Evolution method parameter in MEGA 6 software (Tamura et al., 2013).

## Results and Discussion

### Identification of species and haplotype of prey items by NGS analysis

Before analyzing the stomach contents of *D. mawsoni*, the reliability of the designed universal primer set was validated. A total of 170 metazoan taxa covering 11 phyla were amplified based on NGS analysis of pooled zooplankton samples collected from the Korean coastal waters in 2015, which supported that the newly designed universal COI primer sets would be reliable in the analysis of the stomach contents of *D. mawsoni* (Data S3).

A total of 163 and 164 stomachs were dissected from subareas 58.4 and 88.3, and the average wet weight of contents was 313.52 g ± 387.65 and 351.81 ± 369.89, respectively. From 335,548 and 479,962 raw reads generated from pooled stomach contents from subareas 58.4 and 88.3, 129,931 and 210,089 end-paired contigs were retrieved, respectively. A total of 140,177 contigs from subarea 58.4 and 80,933 contigs from subarea 88.3 were obtained after clustering at 99.6% and discarding the chimeric contigs and those of *D. mawsoni* itself (329 and 129 from subarea 58.4 and 88.3, respectively). A total of 131,233 contigs (130 OTUs) from subarea 58.4 and 75,961 contigs (105 OTUs) from subarea 88.3 were ultimately used for further analysis after low-numbered OTUs (<10) were deleted: 8,944 contigs (6.38%) and 4,972 (6.14%) contigs from area 58.4 and 88.3, respectively, were removed. It was notable that only 0.25% and 0.16% from area 58.4 and 88.3, respectively, of the predator’s own contigs were identified from the stomach contents. It is known that blocking primers should be used to decrease the outnumbered contamination by the predator’s own DNA in molecular diet studies (Leray et al., 2013a; Pompanon et al., 2012; Su et al., 2017). The low amount of the predator’s DNA in this study can be explained by the unique conditions during sample collection. Water temperatures at the sample sites were between 0.7 °C and 0.9 °C and the stomachs of *D. mawsoni* were dissected and stored at below −30 °C immediately after the fish were caught, which may have decreased the chance of contamination of the stomach contents with the predator’s DNA. Further data should be obtained to determine if immediate freezing can lower contamination by the predator’s DNA in other molecular diet studies, in which blocking primers are not required.

All, except four, of the remaining OTUs exhibited 99% identity to the existing sequences in the GenBank database (Datas S4 and S5). Two OTUs exhibited 98% identity to *Chionobathyscus dewitti* and *Lepidonotothen squamifrons*, with contig numbers of 12 and 10, respectively, which is negligibly low. The other two OTUs exhibited 80% and 85% identity with *Peniculus fistula* and *Pseudoterranova bulbosa*, and these were denoted “Unknown-1” and “Unknown-2,” respectively. Remarkably, species names were assigned to more than 98.30% of total OTUs obtained from the stomachs of *D. mawsoni* at 99% sequence identity (Datas S4 and S5). This result may have been obtained because of the high quality of barcode data collected from previous surveys (Smith et al., 2012; Smith et al., 2008), strongly suggesting that the metabarcoding strategy for ecological studies of fish inhabiting the Antarctic Ocean can now be used, with supplementation of only a small amount of additional barcode data.

At 98% sequence identity, a total of 11 and 14 species were identified in the stomachs of *D. mawsoni* collected in subarea 58.4 and 88.3, respectively, excluding “unknown” OTUs (Table 1). Among them, six species were identified in both areas, which consisted of four fish species (*Bathyraja maccaini, Chionobathyscus dewitti, Macrourus whitsoni, Muraenolepis* sp*.*) and mollusks, *Psychroteuthis* sp. and *Cirroctopus* sp. (Table 1). As a result, a total of 19 species (14 fish and 5 mollusks) were identified from the stomachs of *D. mawsoni* in this study (Table 1). Two “Unknown” OTUs belonged to an arthropod and a nematode. The proportion of contigs indicated that more than 90% were fish species, in agreement with previous studies that found the major prey of *D. mawsoni* were fish species (Fenaughty, Stevens & Hanchet, 2003).

**Table 1:** List and proportions of prey items of *Dissostichus mawsoni* as determined by NGS analysis.

Subarea 58.4			Subarea 88.3		
Species	Contigs	%	Species	Contigs	%
*Macrourus whitsoni*	66,262	50.49	*Macrourus whitsoni*	60,707	79.92
*Chionobathyscus dewitti*	44,091	33.60	*Chionobathyscus dewitti*	4,798	6.32
*Muraenolepis sp.*	5,707	4.35	*Lycenchelys sp.*	2,971	3.91
*Anotopterus pharaoh*	4,645	3.54	*Neopagetopsis ionah*	2,030	2.67
*Lepidonotothen squamifrons*	3,166	2.41	*Bathyraja maccaini*	1,291	1.70
*Magnisudis prionosa*	3,076	2.34	*Psychroteuthis sp. (glacialis)*	1,160	1.53
*Muusoctopus (Benthoctopus) levis*	1,958	1.49	*Trematomus lepidorhinus*	950	1.25
*Cirroctopus sp.*	1,025	0.78	*Pogonophryne sp.*-1	568	0.75
*Bathyraja maccaini*	512	0.39	*Pogonophryne scotti*	379	0.50
*Lampris immaculatus*	353	0.27	Unknown-2 (*Nematoda*)	349	0.46
Unknown-1 (*Arthropoda*)	295	0.22	*Cirroctopus sp.*	470	0.62
*Psychroteuthis sp. (glacialis)*	143	0.11	*Muraenolepis sp.*	177	0.23
			*Benthoctopus sp.*	57	0.08
			*Pogonophryne sp.*-2	38	0.05
			*Graneledone antarctica*	16	0.02
Total	131,233	100	Total	75,961	100

To determine if there was a geographical difference between subareas 58.4 and 88.3, we further analyzed the genotypes and the proportion of each assigned species (Table 2). We obtained a total of 35 OTUs as representative haplotypes, which consisted of more than 5% of total OTUs assigned to each species. A total of 18 OTUs were previously reported in the GenBank database, and the other 17 OTUs were newly identified in this study and were deposited in the database as the major haplotypes for the species collected in the Antarctic region (Table 2). Among the 21 assigned species, multiple haplotypes were identified in nine species (Table 2). Four haplotypes were identified for *C. dewitti,* with haplotypes 1 and 3 were already deposited in the GenBank database (GenBank numbers: HQ712909 and JN640824). This species showed distinct geographical differences in haplotypes. Haplotype 1 (GenBank number; HQ712909) was exclusively identified in subarea 58.4, where it was the most dominant genotype of *C. dewitti* (86.82%), whereas haplotype 2 and 3 (GenBank number; KY926829 and JN640824) were the predominant haplotypes in subarea 88.3 (Table 2). Contrary to the results for *C. dewitti*, two haplotypes of *M. whitsoni* were identified in both subareas, but their ratio differed significantly between the areas. The ratio of type 1 and 2 of *M. whitsoni* was 9.34:82.96 and 89.47:13.77 in area 58.4 and 88.3, respectively (Table 2). The geographical difference in genotypes of prey items was most clearly shown in the mollusks, having relatively lower mobility, such as octopus species (Table 2). These results suggest that stomach content analysis using the NGS platform can provide information pertinent to where the fish were caught and more genetic data should be deposited for the better resolution.

**Table 2:** Major genotypes of prey items of *Dissostichus mawsoni*.

Species	Haplotype number	Number of OTUs (Proportions%)		GenBank number	First report in this study^*^
		58.4	88.3		
*Anotopterus vorax* (*pharao*)	1	5 (91.83)		KY926825	O
*Anotopterus vorax* (*pharao*)	2	19 (5.63)		KY926826	O
*Bathyraja maccaini*	1	15 (87.18)	7 (100)	EU119820	
*Bathyraja maccaini*	2	49 (5.13)		KY926827	O
*Bathyraja maccaini*	3	54 (5.13)		KY926828	O
*Muusoctopus (Benthoctopus) levis*	1	9 (98.66)		EF016332	
*Benthoctopus* sp.	1		23 (100)	GU073624	
*Chionobathyscus dewitti*	1	2 (86.82)		HQ712909	
*Chionobathyscus dewitti*	2		3 (59.65)	KY926829	O
*Chionobathyscus dewitti*	3		6 (37.34)	JN640824	
*Chionobathyscus dewitti*	4	6 (6.76)		KY926830	O
*Cirroctopus* sp.	1	10 (100)		KY926831	O
*Cirroctopus* sp.	2		15 (56.45)	GU073528	
*Cirroctopus* sp.	3		20 (29.03)	KY926832	O
*Cirroctopus* sp.	4		22 (14.52)	KY926833	O
*Graneledone antarctica*	1		56 (100)	AF377973	
*Lampris immaculatus*	1	16 (100)		DQ108066	
*Lepidonotothen squamifrons*	1	7 (93.78)		EU326365	
*Lycenchelys* sp.	1		4 (91.07)	JN641010	
*Lycenchelys* sp.	2		16 (8.42)	KY926834	O
*Macrourus whitsoni*	1	3 (9.34)	1 (82.96)	JF265125	
*Macrourus whitsoni*	2	1 (89.47)	2 (13.77)	JF265124	
*Magnisudis prionosa*	1	8 (68.38)		JN640679	
*Magnisudis prionosa*	2	11 (28.63)		KY926835	O
*Muraenolepis* sp.	1	4 (99.31)	17 (100)	HQ713085	
*Neopagetopsis ionah*	1		5 (99.63)	HQ713088	
*Pogonophryne scotti*	2		12 (37.6)	HQ713186	
*Pogonophryne* sp.-1	1		10 (56.8)	KY926837	O
*Pogonophryne sp.*-2	1		31 (100)	KY926836	O
*Psychroteuthis* sp. (*glacialis*)	1	21 (63.64)	8 (98.03)	AY557544	
*Psychroteuthis* sp*.* (*glacialis*)	2	25 (36.36)		KY926838	O
*Trematomus lepidorhinus*	1		9 (57.6)	KY926839	O
*Trematomus lepidorhinus*	2		11 (40.8)	HQ713303	
Unknown-1 (Maxillopoda)	1	17 (100)		KY926840	O
Unknown -2(Chromadorea)	2		14 (100)	KY926841	O

**Notes.**

* 17 newly identified haplotypes in this study are marked with “O”.

### Fish prey items identified by NGS analysis from the stomachs of *D. mawsoni*

**Figure 2:**
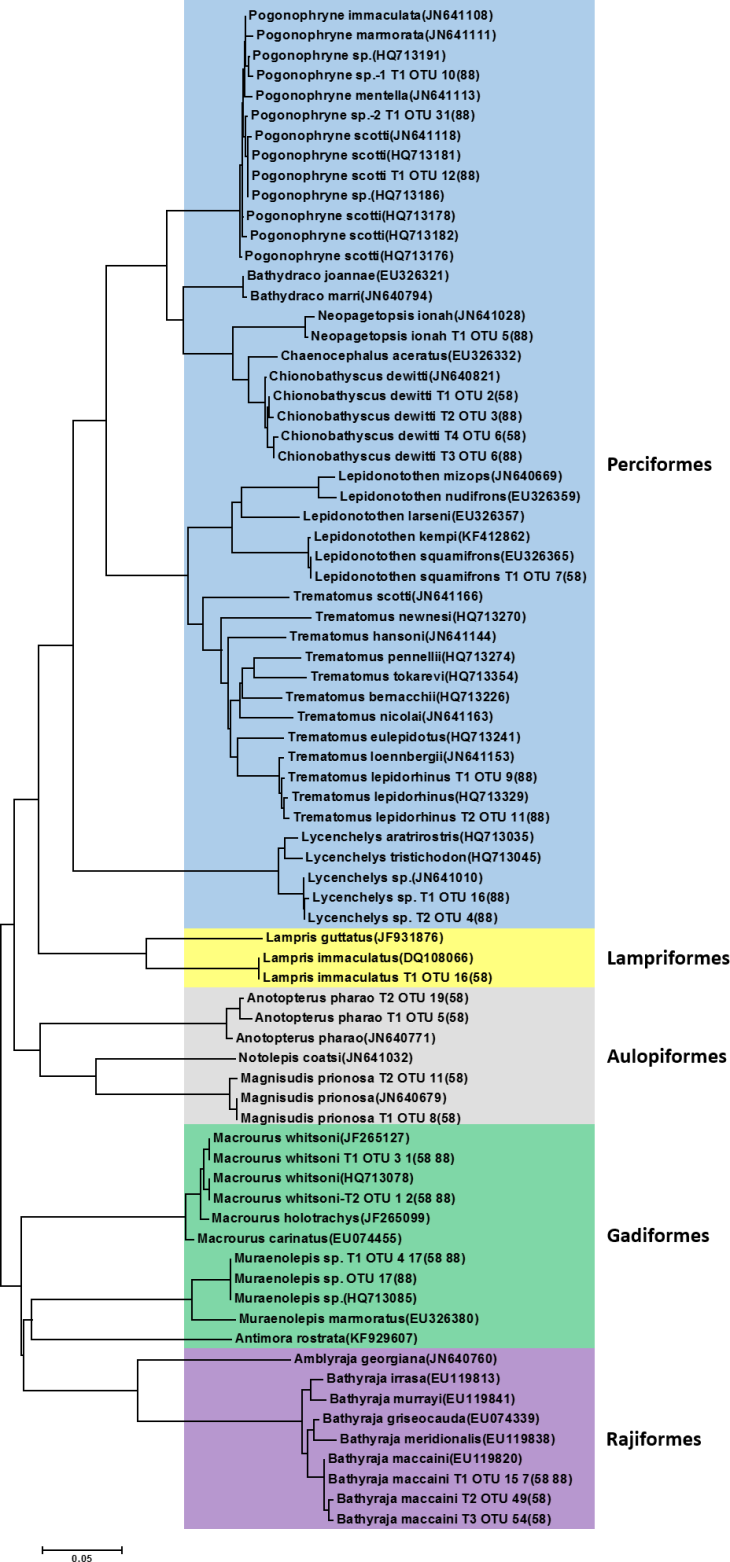
Phylogenetic tree of representative piscine operational taxonomic units (OTUs) identified from the stomach of *Dissostichus mawsoni*. Phylogenetic tree of representative pscine OTUs identified in this study was constructed with vertebrate species identified in previous morphological analysis from the stomach of *Dissostichus mawsoni*. OTUs identified in this study contain information on haplotype number, OTU number, and station of collection.

Fourteen fish OTUs were further analyzed using phylogenetic trees for a comparative analysis between the molecular identification in the present study and results of previous morphological analyses (Fig. 2). The 14 fish prey OTUs were further classified into five orders including eight Perciformes, two Gadiformes, two Aulopiformes, one Lampriformes, and one Rajiformes. The highest number of fish OTUs (eight species) belonged to the order Perciformes, most of which were icefish in the suborder Notothenioidei (Fig. 2). One exceptional OTU exhibited 100% identity to *Lycenchelys* sp. (GenBank Number: JN641010), which belongs to the suborder Zoarcoidei. Currently, 10 species belonging to the genus *Lycenchelys* are reported in the Register of Antarctic Marine Species (RAMS; http://www.marinespecies.org/rams) and only three barcodes are deposited in GenBank, including *Lycenchelys tristichodon* (HQ713045), *Lycenchelys aratrirostris* (HQ713035), *Lycenchelys antarctica* (JN641009). Based on phylogenetic analysis, this OTU was most closely related to *L. tristichodon* (97% sequence identity) (Fig. 2). Because it was only identified in subarea 88.3, further study is needed to identify this species and the geographical distribution of the 10 different *Lycenchelys* species in the Antarctic area. The other seven icefish species in the suborder Notothenioidei were further classified into the families Channichthyidae (*Chionobathyscus dewitti* and *Neopagetopsis ionah*), Nototheniidae (*Lepidonotothen squamifrons* and *Trematomus lepidorhinus*), and Artedidraconidae (*Pogonophryne scotti* and two *Pogonophryne* sp*.*). In addition to *C. dewitti* in the family Channichthyidae, which is well known to be one of the two major fish prey items of *D. mawsoni*, *Neopagetopsis ionah* was also identified in subarea 88.3 (Fig. 2). *Neopagetopsis ionah* was identified only in subarea 88.3 and this is the first report, to our knowledge, that this species is a prey item of *D. mawsoni*. This may have occurred because of its morphological similarity to *C. dewitti*, and the partially digested individuals may not have been easy to be distinguished from the majority of *C. dewitti* in the stomach. This result supports that molecular analysis of stomach contents as the superior tool for the identification of prey items. Two species in the family Nototheniidae, *L. squamifrons* and *Trematomus lepidorhinus*, were also identified only in this study; however, the former was previously reported as *Notothenia kemp* or *L. kempi* (Petrov & Tatarnikov, 2011; Roberts, Xavier & Agnew, 2011) and is now accepted as *L. squamifrons* according to World Register of Marine Species (WoRMS, http://www.marinespecies.org/), and this species was previously reported by its former names. Three OTUs in the family Artedidraconidae belonged to the genus *Pogonophryne* (Fig. 2). According to RAMS, a total of 14 species belonging to the genus *Pogonophryne* (Regan, 1914) are currently verified, and COI data from four species, including *P. marmorata*, *P. mentella*, *P. immaculata*, and *P. scotti* are currently available. The first OTU exhibited 99% identity with *Pogonophryne* sp. (GenBank number: HQ713191) and was denoted *Pogonophryne* sp.-1 (Fig. 2). The second OTU showed 100% sequence identity with *P. scotti* (GenBank number: HQ713181). The third OTU (*Pogonophryne* sp.-2) exhibited 99% sequence identity with three species, including *Pogonophryne immaculate* (GenBank number: JN641108), *Pogonophryne scotti* (GenBank number: Jhq713181), and *Pogonophryne* sp. (GenBank number: HQ713181). These results indicate that the exact molecular identification of samples belonging to the genus *Pogonophryne* is not currently possible, and that the barcode data should be supplemented.

Among the two species belonging to the order Gadiformes, Whitson’s grenadier, *Macrourus whitsoni* (Regan, 1913), was the most important prey item of *D. mawsoni*, which consisted of 49.44% and 80.25% of contigs generated from stomach contents of fish caught in subarea 58.4 and 88.3, respectively (Table 1). This result is consistent with findings of previous studies that reported *M. whitsoni* and *C. dewitti* were the most important prey items of *D. mawsoni*. (Fenaughty, Stevens & Hanchet, 2003; Stevens et al., 2014). The second species belonging to the order Gadiformes was *Muraenolepis* sp. (GenBank number; HQ713085). According to RAMS, three species belonging to the genus *Muraenolepis* (Günther, 1880) have been previously observed in the Antarctic area; *M. marmorata*, *M. microps*, and *M. orangiensis*. Among the three *Muraenolepis* species, only one COI sequence, that of *M. marmorata*, is currently available. The OTU obtained in this study exhibited 100% sequence identity with *Muraenolepis* sp. (HQ713085), which is distinct from *M. marmorata* (Fig. 2). To determine this species, which was identified in both subarea 58.4 and 88.3, further studies are needed.

Two OTUs belonging to order Aulopiformes were that of *Magnisudis prionosa* and *Anotopterus pharaoh* (Fig. 2). The southern barracudina, *M. prionosa* (Rofen, 1963) was exclusively identified by NGS analysis in the present study (Table 3). Although the exact species name was not identified, previous morphological studies also identified barracudina species from the stomachs of *D. mawsoni* (Fenaughty, Stevens & Hanchet, 2003) and the Antarctic jonasfish, *Notolepis coatsi*, belonging to the family Paralepididae (Stevens et al., 2014). Because the two species are morphologically similar to each other and belong to the same family, it is possible that *M. prionosa* was mistakenly identified as *N. coatsi*. In fact, we could not identify the *N. coatsi* sequence in this study. *M. prionosa* exhibited 85% nucleotide sequence identity with *N. coatsi*, which makes them easily distinguishable. *Magnisudis prionosa* was predominantly identified in the *D. mawsoni* caught in subarea 58.4 (Table 1). According to FishBase (http://fishbase.org/), *M. prionosa* is distributed circumglobally in the Southern Ocean from approximately 20°S to Antarctica. Although two OTUs (GenBank Number; KY926825 and KY926826) exhibited the highest sequence identity (99%) to *A. pharaoh* (GenBank Number: EU148072), these OTUs could belong to *A. vorax,* which is currently absent from the Genbank database. Previous morphological analysis identified both *Anotopterus vorax* (Roberts, Xavier & Agnew, 2011) and *A. pharaoh* (Petrov & Tatarnikov, 2011) as prey items of *D. mawsoni*. There are three species in the genus *Anotopterus*, the daggertooth (*A. pharaoh*), the North Pacific daggertooth (*A. nikparini*), and the South Ocean daggertooth (*A. vorax*). The two OTUs exhibited lower sequence identity with *A. nikparini* (98%) and *A. pharaoh* inhabits only the Northern Atlantic Ocean (Templeman, 1970).

One OTU belonging to the order Rajiformes was identified as McCain’s skate, *Bathyraja maccaini* (Springer, 1971). Two previous studies also detected rays as prey items of *D. mawsoni*, but they identified them as the Antarctic starry skate, *Amblyraja georgiana* (Norman, 1938) by morphological analyses (Roberts, Xavier & Agnew, 2011; Stevens et al., 2014). In contrast, we were not able to detect those sequences by NGS analysis. Nucleotide sequence identity in the COI region between the two species is 82%, which makes them easily distinguishable. Although both species were detected primarily in subarea 88.3, *B. maccaini* is more widely distributed than *A. georgiana* in continental waters to the Weddell Sea and eastward, as well as in the Pacific Ocean sector, according to FishBase. In fact, *B. maccaini* was detected in both subarea 58.4 and 88.3, which suggests that this species is a major elasmobranch prey item of *D. mawsoni*, but further study is needed to confirm this. Among the three haplotypes of *B. maccaini* (Springer, 1971), one haplotype, exhibiting 100% identity in the GenBank database (EU119820), occurred at 100% and 87.18% in area 88.3 and 58.4, respectively.

**Table 3:** Comparison of prey items of *Dissostichus mawsoni* determined by morphological and molecular analyses.

Phyla	Species names	A	B	C	D1	D2	Reproducibility
Chordata	*Amblyraja georgiana*		o	o			
	*Anotopterus vorax (pharaoh)*	o		o	o		
	*Antimora rostrata*	o	o	o			
	*Bathydraco joannae*			o			
	*Bathydraco marri*	o					
	*Bathyraja maccaini*				o	o	
	*Chaenocephalus aceratus*			o			
	*Chionobathyscus dewitti*	o			o	o	
	*Lampris immaculatus*				o		
	*Lepidonotothen kempi*	o		o			
	*Lepidonotothen squamifrons*				o		
	*Lycenchelys sp.*					o	
	*Macrourus whitsoni*	o	o	o	o	o	
	*Magnisudis prionosa*				o		
	*Melanocentus rossi*		o				
	*Mesonychoteuthis hamiltoni*	o		o			
	*Muraenolepis microps*	o		o			
	*Muraenolepis sp.*		o		o	o	
	*Neopagetopsis ionah*					o	
	*Notolepis coatsi*		o				
	*Notothenia sp.*			o			
	*Pogonophryne scotti*					o	
	*Pogonophryne sp.*					o	
	*Trematomus lepidorhinus*					o	
Aves	*Pygoscelis antarcticus*			o			
Mollusca	*Alluroteuthis antarcticus*			o			
	*Benthoctopus sp.*					o	
	*Cirroctopus sp.*		o		o	o	
	*Cirroctopus(Grimpoteuthis) antarcticus*	o					
	*Galiteuthis glascialis*			o			
	*Graneledone antarctica*					o	
	*Kondakovia longimana*	o	o	o			
	*Moroteuthis knipovitchi*			o			
	*Muusoctopus (Benthoctopus) levis*				o		
	*Psychroteuthis sp. (glacialis)*		o	o	o	o	
	*Stauroteuthis gilchristi*			o			
Arthropoda	*Nematocarcinus lanceopec*	o					
	*Nematocarcinus sp.*		o	o			
	*Thymops birsteini*			o			
	Unknown (80% identity to *Peniculus fistula*)				o		
	*Eurythenes gryllus*		o				
Annelida	*Nototheniobdella sawyer*					^*^	
Nematoda	Unknown (85% identity to *Pseudoterranova bulbosa*)					o	

**Notes.**

A Petrov & Tatarnikov (2011) B Stevens et al. (2014) C Roberts, Xavier & Agnew (2011) D1 current study in subarea 58.4, and D2 current study in subarea 88.3

* Asterisk indicates OTU with the low contig numbers (<10). Species identified exclusively by morphological analysis, molecular analysis, and both studies were filled with yellow, blue and green color, respectively.

There was one OTU belonging to the order Lampriformes, which was the Southern opah, *Lampris immaculatus* (DQ108066). Two species in the genus *Lampris* are currently known: *L. guttatus* and *L. immaculatus*. *Lampris guttatus* inhabits tropical and temperate ocean waters, whereas *L. immaculatus* is widely distributed in the southern hemisphere between 34°S and the Antarctic Polar Front. *Lampris immaculatus* has not been identified as a prey item of *D. mawsoni* by morphological analysis and they were exclusively identified in subarea 58.4 by NGS analysis (Table 3).

Several species were identified as prey items only by morphological analysis. Two studies identified the blue antimora, *Antimora rostrata* (Günther, 1878), as a prey item (Petrov & Tatarnikov, 2011; Roberts, Xavier & Agnew, 2011), but we failed to detect it in this study. According to FishBase, *A. rostrata* has a wide distribution (62°N–62°S, 180°W–180°E) and is among the most abundant fishes in the abyssal depth ranging from 1,300 and 2,500 m (Iwamoto, 1975; Fossen & Bergstad, 2006). It is mostly observed in the northern hemisphere, such as the North Atlantic, and its genetic population has not been clearly established. Further study should be conducted to determine the contribution of *A. rostrata* to the food web of the Antarctic Ocean. Although *A. georgiana* was identified as a prey item in previous studies (Roberts, Xavier & Agnew, 2011; Stevens et al., 2014), as mentioned above, *B. maccaini* was the only fish species belonging to the class Elasmobranchii detected by molecular analysis. In addition to fish species, remains of a chinstrap penguin, *Pygoscelis antarcticus*, were also identified in a previous morphological study (Stevens et al., 2014), but this species was not found in the NGS results in this study. Fish in the family Bathydraconidae, *Bathydraco joannae* and *B. marri* were also identified only by morphological analysis (Petrov & Tatarnikov, 2011; Roberts, Xavier & Agnew, 2011). Molecular analysis did not detect fish belonging to the family Bathydraconidae. Stevens et al. (2014) also identified *Melanocetus rossi*, which is known to exclusively inhabit the Ross Sea; however, it was not identified in other diet studies or the present study.

**Figure 3:**
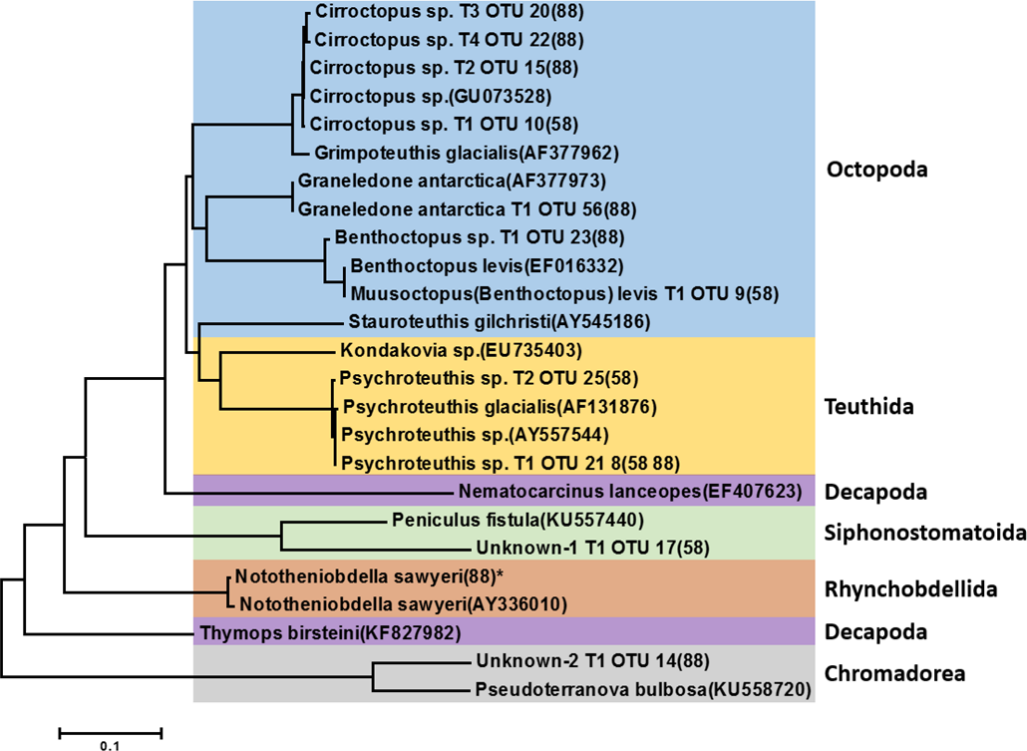
Phylogenetic tree of representative invertebrate operational taxonomic units (OTUs) identified from the stomach of *Dissostichus mawsoni*. Phylogenetic tree of representative invertebrate OTUs identified in this study was constructed with invertebrate species identified in previous morphological analysis from the stomach of *Dissostichus mawsoni*. OTUs identified in this study contain information on haplotype number, OTU number, and station of collection. * indicates the OTU under 10 contig numbers.

### Invertebrate species identified in the stomachs of *D. mawsoni* by molecular analysis

Among 35 representative OTUs as prey items of *D. mawsoni*, 11 belonged to invertebrate species (Table 2). These OTUs were composed of nine mollusks and two unknown taxa (“Unknown-1” and “Unknown-2”). Among the nine molluscan OTUs, seven and two belonged to order Octopoda and order Teuthida, respectively. The Octopoda OTUs could be assigned to four species: *Cirroctopus* sp., *Graneledone antarctica*, *Muusoctopus (Benthoctopus) levis*, and *Benthoctopus* sp. (Fig. 3). The four OTUs (OTU 10 in area 58.4 and OTU 15, 20 and 22 in area 88.3) assigned to *Cirroctopus* sp. (GenBank number: GU073528). According to WoRMs, four species are currently verified in the genus *Cirroctopus*: *Cirroctopus antarcticus* (Kubodera & Okutani, 1986), *C. glacialis* (Robson, 1930), *C. hochbergi* (O’Shea, 1999), and *C. mawsoni* (Berry, 1917). Previous studies identified *Grimpoteuthis antarctica*, but it is now accepted as the same species as *C. antarcticus* (Kubodera & Okutani, 1986). Unfortunately, COI sequences for these species are not currently deposited in the GenBank database and further studies are needed to catalogue them. Another octopus species, *Graneledone antarctica* (Voss, 1976), was identified only in Area 88.3 (OTU 56). One OTU identified only in area 58.4 (OTU 9) exhibited 100% identity with *Benthoctopus levis* (GenBank number: EF016332). According to WoRMs, *B. levis* is now accepted as *Muusoctopus levis* (Hoyle, 1885) and we followed this nomenclature. The other (OTU 23 in Area 88.3) showed 100% identity with *Benthoctopus* sp. (GenBank number: GU073624). *Stauroteuthis gilchristi* was identified by previous morphological analysis (Stevens et al., 2014), but not in this study. Among the two OTUs belonging to the order Teuthida, one OTU showed 100% identity with *Psychroteuthis* sp. (OTU 21 and 8, are 58.4 and 88.3, respectively) (GenBank number: AY557544). The remaining OTUs exhibited 99% identity with the glacial squid, *Psychroteuthis glacialis* (OTU 25 in area 58.4) (GenBank number: AF131876). Because *P. glacialis* is the only species in the monotypic genus *Psychroteuthis* in the family Psychroteuthidae, it appears that these OTUs were *P. glacialis* (Fig. 3). Concerning octopus species, two species previously identified in the order Teuthida, *Kondakovia longimana* and *Mesonychoteuthis hamiltoni* (Petrov & Tatarnikov, 2011; Stevens et al., 2014), were not identified in this study.

We failed to obtain crustacean OTUs from the stomachs of *D. mawsoni*. A commonly identified species in morphological analysis is the Nematocarcinus shrimp (Roberts, Xavier & Agnew, 2011; Stevens et al., 2014). According to the RAMS, only one species, *Nematocarcinus lanceopes* (Spence Bate, 1888), has been reported in the genera for the Antarctic Ocean. In addition, a few decapods (*Thymops birsteini* and *Paralomis* sp.), amphipods, and isopods were detected in the previous studies (Petrov & Tatarnikov, 2011; Stevens et al., 2014). This discrepancy may have been due to the different taxon coverage of the universal primer set used in this study. Although we designed a universal primer set based on the 15,564 COI sequences, which covered 25 metazoan phyla including arthropoda (Datas S1 and S2), it is possible to obtain a different result by the different universal primer set. The other explanation is the different digestion rates for crustacean species. The much longer digestion times needed for hard shells often result in biased evaluation of the importance of prey items (Antonelis et al., 1987; Olson & Boggs, 1986). Remains of exoskeletons of crustaceans may exaggerate their importance as prey items for *D. mawsoni*. Considering the low index of relative importance (% IRI) for the crustacean species (0.01–0.3) (Stevens et al., 2014), it is reasonable not to detect crustacean sequences by NGS analysis.

In addition to prey items, we detected several parasitic OTUs from the stomachs of *D. mawsoni*. The unknown OTU in subarea 58.4 (OTU 17) exhibited 80% nucleotide sequence identity with *Peniculus fistula* and 85% amino acid sequence identity with *Metapeniculus antofagastensis*, which are well known parasites of fishes. The other OTUs in subarea 88.3 (OTU 14) showed 85% identity with *Pseudoterranova bulbosa*, which belongs to the family Anisakidae. In addition, seven contigs encoding the marine leech *Nototheniobdella sawyer* were identified in area 88.4. *N. sawyer* is one of the most commonly identified parasites among Antarctic fish species (Oğuz et al., 2015). Because we only detected the existence of parasites in the stomachs of examined fish by NGS analysis, further study should be conducted on parasitic organisms of *D. mawsoni*, including individual infections, regional differences, and genetic diversity.

### Current status of diet study using the NGS platform and further study

In the present study, we analyzed stomach contents of the Antarctic icefish, *D. mawsoni*, using the Illumina MiSeq platform and compared the data obtained by NGS analysis with those from previous morphological analysis (Table 3). However, collecting *D. mawsoni* samples in Antarctic water is possible only in a limited time and area and we cannot simply compare the reliability of results between the previous morphological analyses and this study. In fact, 92.87% and 88.62% of prey items from in 58.4 and 88.3 area, respectively, were composed of the commonly identified species in all four surveys, including *A. vorax* (*pharaoh*), *C. dewitti*, *M. whitsoni*, *Muraenolepis* sp., *Cirroctopus* sp., and *Psychroteuthis* sp. (*glacialis*) (Table 3). Thus, the results analyzed using the NGS platform and morphological observation were not significantly different. However, NGS studies are characterized by rapid processing time and reliable taxonomic identification for well documented reference databases. In fact, the most significant result in this study was that we could obtain new prey items for *D. mawsoni*, even though their proportions were not as high as the major prey items (Table 3). Nine fish species and three molluscan species were newly identified as prey items of *D. mawsoni* by NGS analysis. This is primarily because of the high quality of DNA barcodes in the GenBank database for fish inhabiting the Antarctic Ocean. Except for several genera, we could identify the species with 98% similarity, which was sufficient to determine each piscine species. According to RAMS, a total of 314 fish species are reported for Antarctica, and molecular identification of stomach contents for *D. mawsoni*, which prey primarily on fish species, was successful. In addition to species identification, genetic diversity of each species could be obtained by NGS analysis. For example, two haplotypes each were obtained for *Macrourus whitsoni*, *Anotopterus vorax*, and *Magnisudis prionosa*, as well as three for *Bathyraja maccaini*, in the present study (Table 3). These data could be further used for the biogeographical study of species in the Antarctic Ocean. Another benefit of molecular analysis is the possibility of reexamination if DNA samples are stored properly. As shown in this study, nomenclature of many species is being changed as molecular phylogeny is changing the traditional taxonomy that was primarily based on morphological characteristics. DNA samples require much smaller storage space than sample bottles. In addition, as better sequencing platforms than those currently used are developed, samples can be reexamined to obtain clearer results.

Although the molecular diet study of *D. mawsoni* was successful in the identification of most prey items, additional efforts are required for the NGS-based diet study to replace the traditionally used morphological analysis. Firstly, DNA barcode data for the invertebrates should be supplemented. Because the prey of *D. mawsoni* was primarily piscine species with high-quality barcode data, we could identify most species simply by DNA sequences. However, we still encountered difficulty in assigning several OTUs with species names in a few fish species and several invertebrates. To construct a better platform for stomach content analysis by NGS, larger barcode databases should be prepared, especially for invertebrates inhabiting both pelagic and benthic areas. As advances occur in high-throughput DNA sequencing technology, soon the stomach contents of fish species inhabiting the Antarctic Ocean will be analyzed simply by DNA sequences, with the supplementation of invertebrate barcode data in the database.

Secondly, we need to develop a reliable method for quantitative analysis of prey items generated by NGS analysis. In the present study, approximately 97% of contigs were fish species, whereas only approximately 3% were invertebrates. Typical quantification of prey items by morphological data are presented as percent frequency of occurrence (%F), percentage by weight (%W), or % IRI. Previous studies showed that the proportion of fish species as prey items of *D. mawsoni* was between 77% and 86% in %F (Fenaughty, Stevens & Hanchet, 2003), between 58% and 62% in % W (Petrov & Tatarnikov, 2011), and between 75% and 95% in % IRI (Stevens et al., 2014). Although it is well known that numbers of contigs generated by NGS represent a rough estimate of the biomass of each species (Saitoh et al., 2016; Yang et al., 2017), there is no currently accepted relationship between traditional measurements and molecular quantification. Because morphological analysis primarily depends on morphological traits of species, it is difficult to quantify the mass of mostly digested stomach contents. In addition, prey items with different digestion times should be considered. Morphological analyses often exaggerate the importance of prey items with hard shells, such as crustaceans, which remain the longest in the stomach, although their cells are completely digested (Jackson, Duffy & Jenkins, 1987; Sheffield et al., 2001). Further studies should be conducted to link morphological and molecular data, for the application of molecular techniques to diet studies of various marine organisms. Finally, primers and PCR conditions also significantly affect metabarcoding results (Berry et al., 2015; Clarke et al., 2017; Harvey et al., 2017) and a reliable universal primer set and a standard protocol for the quantitative NGS analysis of stomach contents should be established. Because the NGS platform has only recently been used in diet studies, primers and sequence protocols vary among different research groups. Although COI universal primers are the most widely used, 18S primers are also often used for their higher taxon coverage. However, their major weakness has been low resolution at the species level (Tang et al., 2012; Zhan et al., 2014). In the present study, we also used a newly developed COI universal primers sets to increase both taxon coverage for PCR and resolution with a longer target region (488–497 bp). This product is longer than those (313 bp) generated by mlCOIintF (Leray et al., 2013b) and jgHCO2198 (Geller et al., 2013), which is one of the most widely used universal primer sets for NGS analyses (Harvey et al., 2017; Leray, Meyer & Mills, 2015; Olmos-Pérez et al., 2017; Yang et al., 2017). Although relatively fewer contigs may be obtained by this primer set compared with those generated by typical COI primers that produce shorter PCR products, we could obtain more useful data not only in species identification, but also in haplotype analysis with much higher resolution. In addition, we also adopted a nested PCR strategy with low cycle numbers to increase the taxon coverage and decrease the PCR-based bias (Yu et al., 2015). Because of the exponential nature of PCR amplification and the variable PCR efficiency of degenerated primers, we could easily identify PCR-based bias, especially in quantification (Caragine et al., 2009; Pinto & Raskin, 2012). As we used the degenerated primer sets, chances of PCR-based bias always exist from the different amplification efficiency of each sequence in the primer. Although nested PCRs with low cycle number may have reduced the bias and increased taxon coverage in present study, reliable quantitative analysis of NGS data is still not completely established and further study should be made.

In conclusion, we adopted the MiSeq platform to analyze the stomach contents of the Antarctic fish, *D. mawsoni*, and compared them with the previous data obtained from morphological analysis. We confirmed that NGS is a reliable technique for the diet study in fish species inhabiting Antarctic water providing useful information with relatively low cost and analysis time. If several limitations are overcome, the NGS technique can be used as a major alternative to the traditional diet study of fish species.

##  Supplemental Information

10.7717/peerj.3977/supp-1 Data S1 Nucleotide sequences of the universal COI primers region and base compositions in the 15,554 species including 25 phyla

10.7717/peerj.3977/supp-2 Data S2 Sequence information for designing the COI universal primer set

10.7717/peerj.3977/supp-3 Data S3 Phylogenetic tree of identified taxa using the newly designed universal COI primer sets Total 170 metazoan taxa were amplified covering 11 phyla as the results of NGS analysis of the pooled zooplankton samples collected from Korean coastal water in 2015. 170 metazoan sequences were aligned using clustal omega (http://www.ebi.ac.uk/Tools/msa/clustalo/). Phylogenetic tree was constructed using the ITOL (itol.embl.de). Phylum is marked in the figure and each color corresponds to a phylum as shown in the legend.

10.7717/peerj.3977/supp-4 Data S4 Description of OTUs obtained from the stomach of *D. mawsoni* in subarea 58.3

10.7717/peerj.3977/supp-5 Data S5 Description of OTUs obtained from the stomach of *D. mawsoni* in subarea 88.4

## Additional Information and Declarations

### DNA Deposition

The following information was supplied regarding the deposition of DNA sequences: The sequences of 17 representative haplotypes newly identified in this study are accessible via Genbank accession numbers from KY926825 to KY926841.

### Data Availability

The following information was supplied regarding data availability: Figshare: https://figshare.com/s/cc379565cf7d23ed29e5.
