# Supplementary material for: Metabarcoding analysis of the stomach contents of the Antarctic Toothfish (Dissostichus mawsoni) collected in the Antarctic Ocean
Source: PeerJ. 2017 Nov 7;5:e3977. doi: 10.7717/peerj.3977 (PMC5680711; doi:10.7717/peerj.3977)
Supplement: Data S1 [file peerj-05-3977-s001.docx]

| **Phylum** | **Align no.** | **COIMISQF1** | **5'** | **A** | **T** | **N** | **G** | **G** | **N** | **G** | **G** | **N** | **T** | **T** | **Y** | **G** | **G** | **N** | **A** | **A** | **3'** |
| --- | --- | --- | --- | --- | --- | --- | --- | --- | --- | --- | --- | --- | --- | --- | --- | --- | --- | --- | --- | --- | --- |
| Acanthocepha | 6 |  |  | 100% | 100% | 100% | 100% | 100% | 100% | 100% | 100% | 100% | 100% | 100% | 100% | 100% | 100% | 100% | 100% | 100% |  |
| Acoelomorpha / Xenacoelomorpha | 6 |  |  | 100% | 83% | 100% | 100% | 100% | 100% | 100% | 100% | 100% | 100% | 100% | 100% | 100% | 100% | 100% | 100% | 100% |  |
| Annelida | 344 |  |  | 96% | 100% | 100% | 99% | 100% | 100% | 100% | 99% | 100% | 100% | 100% | 100% | 100% | 100% | 100% | 100% | 100% |  |
| Arthropoda | 7,140 |  |  | 99% | 100% | 100% | 100% | 100% | 100% | 100% | 100% | 100% | 100% | 100% | 100% | 100% | 100% | 100% | 100% | 100% |  |
| Brachiopoda | 31 |  |  | 100% | 100% | 100% | 100% | 100% | 100% | 100% | 97% | 100% | 97% | 100% | 94% | 100% | 100% | 100% | 100% | 100% |  |
| Bryozoa | 15 |  |  | 93% | 100% | 100% | 100% | 100% | 100% | 100% | 100% | 100% | 100% | 100% | 100% | 100% | 100% | 100% | 100% | 100% |  |
| Chaetognatha | 11 |  |  | 100% | 100% | 100% | 100% | 100% | 100% | 100% | 100% | 100% | 100% | 100% | 100% | 100% | 100% | 100% | 100% | 100% |  |
| Chordata | 6,370 |  |  | 100% | 100% | 100% | 100% | 100% | 100% | 100% | 100% | 100% | 100% | 100% | 100% | 100% | 100% | 100% | 100% | 100% |  |
| Cnidaria | 387 |  |  | 99% | 100% | 100% | 100% | 100% | 100% | 100% | 100% | 100% | 100% | 96% | 100% | 100% | 100% | 100% | 100% | 100% |  |
| Ctenophora | 12 |  |  | 100% | 100% | 100% | 100% | 100% | 100% | 100% | 100% | 100% | 83% | 100% | 8% | 75% | 75% | 100% | 100% | 100% |  |
| Cycliophora |  |  |  | - | - | - | - | - | - | - | - | - | - | - | - | - | - | - | - | - |  |
| Dicyemida / Rhombozoa |  |  |  | - | - | - | - | - | - | - | - | - | - | - | - | - | - | - | - | - |  |
| Echinodermata | 417 |  |  | 100% | 100% | 100% | 100% | 100% | 100% | 100% | 100% | 100% | 100% | 100% | 100% | 100% | 100% | 100% | 100% | 100% |  |
| Entoprocta | 3 |  |  | 100% | 100% | 100% | 100% | 100% | 100% | 100% | 100% | 100% | 100% | 100% | 100% | 100% | 100% | 100% | 100% | 100% |  |
| Gastrotricha | 6 |  |  | 83% | 100% | 100% | 100% | 100% | 100% | 100% | 100% | 100% | 100% | 100% | 100% | 100% | 100% | 100% | 100% | 100% |  |
| Gnathostomulida |  |  |  | - | - | - | - | - | - | - | - | - | - | - | - | - | - | - | - | - |  |
| Hemichordata | 2 |  |  | 100% | 100% | 100% | 100% | 100% | 100% | 100% | 100% | 100% | 100% | 100% | 100% | 100% | 100% | 100% | 100% | 100% |  |
| Kinorhyncha |  |  |  | - | - | - | - | - | - | - | - | - | - | - | - | - | - | - | - | - |  |
| Loricifera |  |  |  | - | - | - | - | - | - | - | - | - | - | - | - | - | - | - | - | - |  |
| Micrognathozoa |  |  |  | - | - | - | - | - | - | - | - | - | - | - | - | - | - | - | - | - |  |
| Mollusca | 292 |  |  | 96% | 100% | 100% | 100% | 100% | 100% | 100% | 100% | 100% | 100% | 100% | 100% | 100% | 100% | 100% | 100% | 100% |  |
| Nematoda | 122 |  |  | 95% | 100% | 100% | 100% | 100% | 100% | 100% | 98% | 100% | 99% | 100% | 99% | 100% | 100% | 100% | 100% | 100% |  |
| Nematomorpha |  |  |  | - | - | - | - | - | - | - | - | - | - | - | - | - | - | - | - | - |  |
| Nemertea | 27 |  |  | 100% | 100% | 100% | 100% | 100% | 100% | 100% | 100% | 100% | 100% | 100% | 100% | 100% | 100% | 100% | 100% | 100% |  |
| Onychophora | 46 |  |  | 100% | 100% | 100% | 100% | 100% | 100% | 100% | 100% | 100% | 100% | 100% | 100% | 100% | 100% | 100% | 100% | 100% |  |
| Orthonectida |  |  |  | - | - | - | - | - | - | - | - | - | - | - | - | - | - | - | - | - |  |
| Phoronida | 3 |  |  | 100% | 100% | 100% | 100% | 100% | 100% | 100% | 100% | 100% | 100% | 100% | 100% | 100% | 100% | 100% | 100% | 100% |  |
| Placozoa |  |  |  | - | - | - | - | - | - | - | - | - | - | - | - | - | - | - | - | - |  |
| Platyhelminthes | 133 |  |  | 97% | 100% | 100% | 100% | 100% | 100% | 100% | 100% | 100% | 100% | 100% | 100% | 100% | 100% | 100% | 100% | 100% |  |
| Porifera | 131 |  |  | 100% | 100% | 100% | 100% | 100% | 100% | 97% | 100% | 100% | 100% | 100% | 100% | 100% | 100% | 100% | 100% | 100% |  |
| Priapulida | 2 |  |  | 100% | 100% | 100% | 100% | 100% | 100% | 100% | 100% | 100% | 100% | 100% | 100% | 100% | 100% | 100% | 100% | 100% |  |
| Rotifera | 20 |  |  | 55% | 100% | 100% | 100% | 100% | 100% | 100% | 100% | 100% | 100% | 100% | 100% | 100% | 100% | 100% | 100% | 100% |  |
| Sipuncula | 15 |  |  | 100% | 100% | 100% | 100% | 100% | 100% | 100% | 100% | 100% | 100% | 100% | 100% | 100% | 100% | 100% | 100% | 100% |  |
| Tardigrada | 23 |  |  | 100% | 100% | 100% | 100% | 100% | 100% | 100% | 100% | 100% | 100% | 100% | 100% | 100% | 100% | 100% | 100% | 100% |  |
| Total | 15,564 |  |  |  |  |  |  |  |  |  |  |  |  |  |  |  |  |  |  |  |  |

| **Phylum** | **Align no.** | **COIMISQF2** | **5'** | **G** | **G** | **N** | **G** | **G** | **N** | **T** | **T** | **Y** | **G** | **G** | **N** | **A** | **A** | **Y** | **T** | **G** | **3'** |
| --- | --- | --- | --- | --- | --- | --- | --- | --- | --- | --- | --- | --- | --- | --- | --- | --- | --- | --- | --- | --- | --- |
| Acanthocepha | 6 |  |  | 100% | 100% | 100% | 100% | 100% | 100% | 100% | 100% | 100% | 100% | 100% | 100% | 100% | 100% | 100% | 100% | 100% |  |
| Acoelomorpha / Xenacoelomorpha | 6 |  |  | 100% | 100% | 100% | 100% | 100% | 100% | 100% | 100% | 100% | 100% | 100% | 100% | 100% | 100% | 100% | 100% | 100% |  |
| Annelida | 344 |  |  | 99% | 100% | 100% | 100% | 99% | 100% | 100% | 100% | 100% | 100% | 100% | 100% | 100% | 100% | 100% | 100% | 99% |  |
| Arthropoda | 7,140 |  |  | 100% | 100% | 100% | 100% | 100% | 100% | 100% | 100% | 100% | 100% | 100% | 100% | 100% | 100% | 100% | 100% | 94% |  |
| Brachiopoda | 31 |  |  | 100% | 100% | 100% | 100% | 97% | 100% | 97% | 100% | 94% | 100% | 100% | 100% | 100% | 100% | 100% | 100% | 100% |  |
| Bryozoa | 15 |  |  | 100% | 100% | 100% | 100% | 100% | 100% | 100% | 100% | 100% | 100% | 100% | 100% | 100% | 100% | 100% | 100% | 100% |  |
| Chaetognatha | 11 |  |  | 100% | 100% | 100% | 100% | 100% | 100% | 100% | 100% | 100% | 100% | 100% | 100% | 100% | 100% | 100% | 100% | 100% |  |
| Chordata | 6,370 |  |  | 100% | 100% | 100% | 100% | 100% | 100% | 100% | 100% | 100% | 100% | 100% | 100% | 100% | 100% | 100% | 100% | 100% |  |
| Cnidaria | 387 |  |  | 100% | 100% | 100% | 100% | 100% | 100% | 100% | 96% | 100% | 100% | 100% | 100% | 100% | 100% | 100% | 100% | 99% |  |
| Ctenophora | 12 |  |  | 100% | 100% | 100% | 100% | 100% | 100% | 83% | 100% | 8% | 75% | 75% | 100% | 100% | 100% | 100% | 100% | 75% |  |
| Cycliophora |  |  |  | - | - | - | - | - | - | - | - | - | - | - | - | - | - | - | - | - |  |
| Dicyemida / Rhombozoa |  |  |  | - | - | - | - | - | - | - | - | - | - | - | - | - | - | - | - | - |  |
| Echinodermata | 417 |  |  | 100% | 100% | 100% | 100% | 100% | 100% | 100% | 100% | 100% | 100% | 100% | 100% | 100% | 100% | 71% | 100% | 100% |  |
| Entoprocta | 3 |  |  | 100% | 100% | 100% | 100% | 100% | 100% | 100% | 100% | 100% | 100% | 100% | 100% | 100% | 100% | 100% | 100% | 100% |  |
| Gastrotricha | 6 |  |  | 100% | 100% | 100% | 100% | 100% | 100% | 100% | 100% | 100% | 100% | 100% | 100% | 100% | 100% | 100% | 83% | 100% |  |
| Gnathostomulida |  |  |  | - | - | - | - | - | - | - | - | - | - | - | - | - | - | - | - | - |  |
| Hemichordata | 2 |  |  | 100% | 100% | 100% | 100% | 100% | 100% | 100% | 100% | 100% | 100% | 100% | 100% | 100% | 100% | 100% | 100% | 100% |  |
| Kinorhyncha |  |  |  | - | - | - | - | - | - | - | - | - | - | - | - | - | - | - | - | - |  |
| Loricifera |  |  |  | - | - | - | - | - | - | - | - | - | - | - | - | - | - | - | - | - |  |
| Micrognathozoa |  |  |  | - | - | - | - | - | - | - | - | - | - | - | - | - | - | - | - | - |  |
| Mollusca | 292 |  |  | 100% | 100% | 100% | 100% | 100% | 100% | 100% | 100% | 100% | 100% | 100% | 100% | 100% | 100% | 100% | 100% | 99% |  |
| Nematoda | 122 |  |  | 100% | 100% | 100% | 100% | 98% | 100% | 99% | 100% | 99% | 100% | 100% | 100% | 100% | 100% | 100% | 99% | 89% |  |
| Nematomorpha |  |  |  | - | - | - | - | - | - | - | - | - | - | - | - | - | - | - | - | - |  |
| Nemertea | 27 |  |  | 100% | 100% | 100% | 100% | 100% | 100% | 100% | 100% | 100% | 100% | 100% | 100% | 100% | 100% | 100% | 100% | 100% |  |
| Onychophora | 46 |  |  | 100% | 100% | 100% | 100% | 100% | 100% | 100% | 100% | 100% | 100% | 100% | 100% | 100% | 100% | 100% | 100% | 100% |  |
| Orthonectida |  |  |  | - | - | - | - | - | - | - | - | - | - | - | - | - | - | - | - | - |  |
| Phoronida | 3 |  |  | 100% | 100% | 100% | 100% | 100% | 100% | 100% | 100% | 100% | 100% | 100% | 100% | 100% | 100% | 100% | 100% | 100% |  |
| Placozoa |  |  |  | - | - | - | - | - | - | - | - | - | - | - | - | - | - | - | - | - |  |
| Platyhelminthes | 133 |  |  | 100% | 100% | 100% | 100% | 100% | 100% | 100% | 100% | 100% | 100% | 100% | 100% | 100% | 100% | 68% | 100% | 55% |  |
| Porifera | 131 |  |  | 100% | 100% | 100% | 97% | 100% | 100% | 100% | 100% | 100% | 100% | 100% | 100% | 100% | 100% | 100% | 100% | 100% |  |
| Priapulida | 2 |  |  | 100% | 100% | 100% | 100% | 100% | 100% | 100% | 100% | 100% | 100% | 100% | 100% | 100% | 100% | 100% | 100% | 100% |  |
| Rotifera | 20 |  |  | 100% | 100% | 100% | 100% | 100% | 100% | 100% | 100% | 100% | 100% | 100% | 100% | 100% | 100% | 100% | 100% | 100% |  |
| Sipuncula | 15 |  |  | 100% | 100% | 100% | 100% | 100% | 100% | 100% | 100% | 100% | 100% | 100% | 100% | 100% | 100% | 100% | 100% | 100% |  |
| Tardigrada | 23 |  |  | 100% | 100% | 100% | 100% | 100% | 100% | 100% | 100% | 100% | 100% | 100% | 100% | 100% | 100% | 100% | 100% | 100% |  |
| Total | 15,564 |  |  |  |  |  |  |  |  |  |  |  |  |  |  |  |  |  |  |  |  |

| **Phylum** | **Align no.** | **Complement sequences of COIMISQR1** | **5'** | **G** | **G** | **N** | **C** | **A** | **Y** | **C** | **C** | **N** | **G** | **A** | **R** | **G** | **T** | **N** | **T** | **A** | **3'** |
| --- | --- | --- | --- | --- | --- | --- | --- | --- | --- | --- | --- | --- | --- | --- | --- | --- | --- | --- | --- | --- | --- |
|  |  |  | **3'** | **C** | **C** | **N** | **G** | **T** | **R** | **G** | **G** | **N** | **C** | **T** | **Y** | **C** | **A** | **N** | **A** | **T** | **5'** |
| Acanthocepha | 6 |  |  | 100% | 100% | 100% | 100% | 100% | 100% | 100% | 100% | 100% | 100% | 100% | 100% | 0% | 0% | 100% | 100% | 0% |  |
| Acoelomorpha / Xenacoelomorpha | 6 |  |  | 100% | 100% | 100% | 100% | 100% | 100% | 100% | 100% | 100% | 100% | 100% | 100% | 100% | 100% | 100% | 100% | 100% |  |
| Annelida | 344 |  |  | 100% | 100% | 100% | 100% | 100% | 100% | 100% | 98% | 100% | 99% | 98% | 100% | 85% | 87% | 100% | 99% | 88% |  |
| Arthropoda | 7,140 |  |  | 100% | 100% | 100% | 100% | 100% | 100% | 100% | 100% | 100% | 100% | 99% | 100% | 99% | 99% | 100% | 100% | 99% |  |
| Brachiopoda | 31 |  |  | 100% | 100% | 100% | 100% | 100% | 100% | 100% | 100% | 100% | 100% | 100% | 100% | 100% | 100% | 100% | 100% | 100% |  |
| Bryozoa | 15 |  |  | 100% | 100% | 100% | 100% | 93% | 100% | 100% | 93% | 100% | 93% | 100% | 100% | 75% | 100% | 100% | 100% | 100% |  |
| Chaetognatha | 11 |  |  | 100% | 100% | 100% | 100% | 100% | 100% | 100% | 100% | 100% | 100% | 100% | 100% | 100% | 100% | 100% | 100% | 100% |  |
| Chordata | 6,370 |  |  | 100% | 100% | 100% | 100% | 100% | 100% | 100% | 100% | 100% | 100% | 100% | 100% | 100% | 100% | 100% | 100% | 100% |  |
| Cnidaria | 387 |  |  | 100% | 100% | 100% | 100% | 100% | 100% | 100% | 100% | 100% | 99% | 98% | 100% | 96% | 96% | 100% | 100% | 97% |  |
| Ctenophora | 12 |  |  | 100% | 100% | 100% | 100% | 100% | 100% | 100% | 100% | 100% | 100% | 100% | 100% | 100% | 100% | 100% | 100% | 100% |  |
| Cycliophora |  |  |  | - | - | - | - | - | - | - | - | - | - | - | - | - | - | - | - | - |  |
| Dicyemida / Rhombozoa |  |  |  | - | - | - | - | - | - | - | - | - | - | - | - | - | - | - | - | - |  |
| Echinodermata | 417 |  |  | 100% | 100% | 100% | 100% | 100% | 100% | 100% | 100% | 100% | 100% | 100% | 100% | 100% | 100% | 100% | 100% | 100% |  |
| Entoprocta | 3 |  |  | 100% | 100% | 100% | 100% | 100% | 100% | 100% | 100% | 100% | 100% | 100% | 100% | 100% | 100% | 100% | 100% | 100% |  |
| Gastrotricha | 6 |  |  | 100% | 100% | 100% | 100% | 100% | 100% | 100% | 100% | 100% | 100% | 100% | 100% | 100% | 100% | 100% | 100% | 100% |  |
| Gnathostomulida |  |  |  | - | - | - | - | - | - | - | - | - | - | - | - | - | - | - | - | - |  |
| Hemichordata | 2 |  |  | 100% | 100% | 100% | 100% | 100% | 100% | 100% | 100% | 100% | 100% | 100% | 100% | 100% | 100% | 100% | 100% | 100% |  |
| Kinorhyncha |  |  |  | - | - | - | - | - | - | - | - | - | - | - | - | - | - | - | - | - |  |
| Loricifera |  |  |  | - | - | - | - | - | - | - | - | - | - | - | - | - | - | - | - | - |  |
| Micrognathozoa |  |  |  | - | - | - | - | - | - | - | - | - | - | - | - | - | - | - | - | - |  |
| Mollusca | 292 |  |  | 100% | 100% | 100% | 100% | 100% | 100% | 100% | 99% | 100% | 99% | 98% | 100% | 97% | 97% | 100% | 100% | 98% |  |
| Nematoda | 122 |  |  | 100% | 100% | 100% | 100% | 100% | 100% | 100% | 100% | 100% | 100% | 100% | 100% | 100% | 100% | 100% | 100% | 100% |  |
| Nematomorpha |  |  |  | - | - | - | - | - | - | - | - | - | - | - | - | - | - | - | - | - |  |
| Nemertea | 27 |  |  | 100% | 100% | 100% | 100% | 100% | 100% | 100% | 100% | 100% | 100% | 100% | 100% | 100% | 100% | 100% | 100% | 100% |  |
| Onychophora | 46 |  |  | 100% | 100% | 100% | 100% | 100% | 100% | 100% | 100% | 100% | 100% | 100% | 100% | 100% | 100% | 100% | 100% | 100% |  |
| Orthonectida |  |  |  | - | - | - | - | - | - | - | - | - | - | - | - | - | - | - | - | - |  |
| Phoronida | 3 |  |  | 100% | 100% | 100% | 100% | 100% | 100% | 100% | 100% | 100% | 100% | 100% | 100% | 100% | 100% | 100% | 100% | 100% |  |
| Placozoa |  |  |  | - | - | - | - | - | - | - | - | - | - | - | - | - | - | - | - | - |  |
| Platyhelminthes | 133 |  |  | 100% | 100% | 100% | 100% | 100% | 100% | 100% | 100% | 100% | 100% | 100% | 100% | 100% | 100% | 100% | 100% | 100% |  |
| Porifera | 131 |  |  | 100% | 100% | 100% | 100% | 100% | 100% | 100% | 100% | 100% | 100% | 100% | 100% | 100% | 100% | 100% | 100% | 100% |  |
| Priapulida | 2 |  |  | 100% | 100% | 100% | 100% | 100% | 100% | 100% | 100% | 100% | 100% | 100% | 100% | 100% | 100% | 100% | 100% | 100% |  |
| Rotifera | 20 |  |  | 100% | 100% | 100% | 100% | 100% | 100% | 100% | 100% | 100% | 94% | 100% | 100% | 100% | 100% | 100% | 100% | 100% |  |
| Sipuncula | 15 |  |  | 100% | 100% | 100% | 100% | 100% | 100% | 100% | 100% | 100% | 100% | 100% | 100% | 100% | 100% | 100% | 100% | 100% |  |
| Tardigrada | 23 |  |  | 100% | 100% | 100% | 96% | 96% | 96% | 100% | 96% | 100% | 95% | 100% | 100% | 95% | 100% | 100% | 95% | 100% |  |
| Total | 15,564 |  |  |  |  |  |  |  |  |  |  |  |  |  |  |  |  |  |  |  |  |

| **Phylum** | **Align no.** | **Complement sequences of COIMISQR2** | **5'** | **T** | **G** | **R** | **T** | **T** | **Y** | **T** | **T** | **Y** | **G** | **G** | **N** | **C** | **A** | **Y** | **C** | **C** | **3'** |
| --- | --- | --- | --- | --- | --- | --- | --- | --- | --- | --- | --- | --- | --- | --- | --- | --- | --- | --- | --- | --- | --- |
|  |  |  | **3'** | **A** | **C** | **Y** | **A** | **A** | **R** | **A** | **A** | **R** | **C** | **C** | **N** | **G** | **T** | **R** | **G** | **G** | **5'** |
| Acanthocepha | 6 |  |  | 100% | 100% | 100% | 100% | 100% | 100% | 100% | 100% | 100% | 100% | 100% | 100% | 100% | 100% | 100% | 100% | 100% |  |
| Acoelomorpha / Xenacoelomorpha | 6 |  |  | 100% | 100% | 100% | 100% | 100% | 100% | 100% | 100% | 100% | 100% | 100% | 100% | 100% | 100% | 100% | 100% | 100% |  |
| Annelida | 344 |  |  | 100% | 100% | 100% | 100% | 100% | 100% | 100% | 99% | 100% | 100% | 100% | 100% | 100% | 100% | 100% | 100% | 98% |  |
| Arthropoda | 7,140 |  |  | 100% | 100% | 100% | 100% | 100% | 100% | 100% | 100% | 100% | 100% | 100% | 100% | 100% | 100% | 100% | 100% | 100% |  |
| Brachiopoda | 31 |  |  | 100% | 100% | 100% | 100% | 100% | 100% | 100% | 100% | 97% | 100% | 100% | 100% | 100% | 100% | 100% | 100% | 100% |  |
| Bryozoa | 15 |  |  | 100% | 100% | 100% | 100% | 100% | 100% | 100% | 100% | 100% | 100% | 100% | 100% | 100% | 93% | 100% | 100% | 93% |  |
| Chaetognatha | 11 |  |  | 100% | 100% | 100% | 100% | 100% | 100% | 100% | 100% | 100% | 100% | 100% | 100% | 100% | 100% | 100% | 100% | 100% |  |
| Chordata | 6,370 |  |  | 100% | 100% | 100% | 100% | 100% | 100% | 100% | 100% | 100% | 100% | 100% | 100% | 100% | 100% | 100% | 100% | 100% |  |
| Cnidaria | 387 |  |  | 100% | 100% | 100% | 100% | 100% | 100% | 100% | 100% | 100% | 100% | 100% | 100% | 100% | 100% | 100% | 100% | 100% |  |
| Ctenophora | 12 |  |  | 100% | 100% | 100% | 100% | 100% | 100% | 100% | 100% | 100% | 100% | 100% | 100% | 100% | 100% | 100% | 100% | 100% |  |
| Cycliophora |  |  |  | - | - | - | - | - | - | - | - | - | - | - | - | - | - | - | - | - |  |
| Dicyemida / Rhombozoa |  |  |  | - | - | - | - | - | - | - | - | - | - | - | - | - | - | - | - | - |  |
| Echinodermata | 417 |  |  | 100% | 100% | 100% | 100% | 100% | 100% | 100% | 100% | 100% | 100% | 100% | 100% | 100% | 100% | 100% | 100% | 100% |  |
| Entoprocta | 3 |  |  | 100% | 100% | 100% | 100% | 100% | 100% | 100% | 100% | 100% | 100% | 100% | 100% | 100% | 100% | 100% | 100% | 100% |  |
| Gastrotricha | 6 |  |  | 100% | 100% | 100% | 100% | 100% | 100% | 100% | 100% | 100% | 100% | 100% | 100% | 100% | 100% | 100% | 100% | 100% |  |
| Gnathostomulida |  |  |  | - | - | - | - | - | - | - | - | - | - | - | - | - | - | - | - | - |  |
| Hemichordata | 2 |  |  | 100% | 100% | 100% | 100% | 100% | 100% | 100% | 100% | 100% | 100% | 100% | 100% | 100% | 100% | 100% | 100% | 100% |  |
| Kinorhyncha |  |  |  | - | - | - | - | - | - | - | - | - | - | - | - | - | - | - | - | - |  |
| Loricifera |  |  |  | - | - | - | - | - | - | - | - | - | - | - | - | - | - | - | - | - |  |
| Micrognathozoa |  |  |  | - | - | - | - | - | - | - | - | - | - | - | - | - | - | - | - | - |  |
| Mollusca | 292 |  |  | 100% | 99% | 100% | 100% | 100% | 100% | 100% | 100% | 100% | 100% | 100% | 100% | 100% | 100% | 100% | 100% | 99% |  |
| Nematoda | 122 |  |  | 99% | 99% | 99% | 100% | 100% | 100% | 100% | 100% | 100% | 100% | 100% | 100% | 100% | 100% | 100% | 100% | 100% |  |
| Nematomorpha |  |  |  | - | - | - | - | - | - | - | - | - | - | - | - | - | - | - | - | - |  |
| Nemertea | 27 |  |  | 100% | 100% | 100% | 100% | 100% | 100% | 100% | 100% | 100% | 100% | 100% | 100% | 100% | 100% | 100% | 100% | 100% |  |
| Onychophora | 46 |  |  | 100% | 100% | 100% | 100% | 100% | 100% | 100% | 100% | 100% | 100% | 100% | 100% | 100% | 100% | 100% | 100% | 100% |  |
| Orthonectida |  |  |  | - | - | - | - | - | - | - | - | - | - | - | - | - | - | - | - | - |  |
| Phoronida | 3 |  |  | 100% | 100% | 100% | 100% | 100% | 100% | 100% | 100% | 100% | 100% | 100% | 100% | 100% | 100% | 100% | 100% | 100% |  |
| Placozoa |  |  |  | - | - | - | - | - | - | - | - | - | - | - | - | - | - | - | - | - |  |
| Platyhelminthes | 133 |  |  | 100% | 100% | 100% | 100% | 100% | 100% | 99% | 100% | 100% | 100% | 100% | 100% | 100% | 100% | 100% | 100% | 100% |  |
| Porifera | 131 |  |  | 99% | 100% | 100% | 100% | 100% | 100% | 100% | 100% | 100% | 100% | 100% | 100% | 100% | 100% | 100% | 100% | 100% |  |
| Priapulida | 2 |  |  | 100% | 100% | 100% | 100% | 100% | 100% | 100% | 100% | 100% | 100% | 100% | 100% | 100% | 100% | 100% | 100% | 100% |  |
| Rotifera | 20 |  |  | 100% | 100% | 100% | 100% | 100% | 100% | 100% | 95% | 100% | 100% | 100% | 100% | 100% | 100% | 100% | 100% | 100% |  |
| Sipuncula | 15 |  |  | 100% | 100% | 100% | 100% | 100% | 100% | 100% | 100% | 100% | 100% | 100% | 100% | 100% | 100% | 100% | 100% | 100% |  |
| Tardigrada | 23 |  |  | 100% | 100% | 100% | 100% | 100% | 100% | 100% | 100% | 100% | 100% | 100% | 100% | 96% | 96% | 96% | 100% | 96% |  |
| Total | 15,564 |  |  |  |  |  |  |  |  |  |  |  |  |  |  |  |  |  |  |  |  |
