# Supplementary material for: Metabarcoding analysis of the stomach contents of the Antarctic Toothfish (Dissostichus mawsoni) collected in the Antarctic Ocean
Source: PeerJ. 2017 Nov 7;5:e3977. doi: 10.7717/peerj.3977 (PMC5680711; doi:10.7717/peerj.3977)
Supplement: Data S4 [file peerj-05-3977-s004.docx]

| OTU | Species | GenBank | Identity | contigs |
| --- | --- | --- | --- | --- |
| OTU_1 | *Macrourus whitsoni* | JF265124 | 100% | 59283 |
| OTU_2 | *Chionobathyscus dewitti* | HQ712909 | 100% | 38279 |
| OTU_3 | *Macrourus whitsoni* | JF265125 | 100% | 6190 |
| OTU_4 | *Muraenolepis sp.* | HQ713085 | 100% | 5662 |
| OTU_5 | *Anotopterus pharaoh* | EU148072 | 99% | 4274 |
| OTU_6 | *Chionobathyscus dewitti* | JN640824 | 99% | 2974 |
| OTU_7 | *Lepidonotothen squamifrons* | EU326365 | 100% | 2965 |
| OTU_8 | *Magnisudis prionosa* | JN640679 | 100% | 2100 |
| OTU_9 | *Muusoctopus (Benthoctopus) levis* | EF016332 | 100% | 1933 |
| OTU_10 | *Cirroctopus sp.* | GU073528 | 99% | 1025 |
| OTU_11 | *Magnisudis prionosa* | JN640679 | 99% | 875 |
| OTU_12 | *Chionobathyscus dewitti* | HQ712909 | 99% | 711 |
| OTU_13 | *Chionobathyscus dewitti* | JN640815 | 100% | 474 |
| OTU_14 | *Chionobathyscus dewitti* | HQ712909 | 99% | 451 |
| OTU_15 | *Bathyraja maccaini* | EU119820 | 100% | 451 |
| OTU_16 | *Lampris immaculatus* | DQ108066 | 100% | 353 |
| OTU_17 | Unknown | KU557422 | 80% | 295 |
| OTU_18 | *Macrourus whitsoni* | JF265124 | 99% | 286 |
| OTU_19 | *Anotopterus pharaoh* | EU148072 | 99% | 261 |
| OTU_20 | *Lepidonotothen squamifrons* | EU326368 | 99% | 122 |
| OTU_21 | *Psychroteuthis sp. (glacialis)* | AY557544 | 100% | 86 |
| OTU_22 | *Chionobathyscus dewitti* | HQ712909 | 99% | 71 |
| OTU_23 | *Macrourus whitsoni* | JF265124 | 99% | 63 |
| OTU_24 | *Macrourus whitsoni* | JF265124 | 99% | 62 |
| OTU_25 | *Psychroteuthis sp. (glacialis)* | AY557544 | 99% | 57 |
| OTU_26 | *Anotopterus pharaoh* | KF929603 | 99% | 56 |
| OTU_27 | *Chionobathyscus dewitti* | JN640824 | 99% | 54 |
| OTU_28 | *Chionobathyscus dewitti* | HQ712909 | 99% | 49 |
| OTU_29 | *Chionobathyscus dewitti* | HQ712909 | 99% | 47 |
| OTU_30 | *Chionobathyscus dewitti* | HQ712909 | 99% | 43 |
| OTU_31 | *Chionobathyscus dewitti* | HQ712909 | 99% | 42 |
| OTU_32 | *Magnisudis prionosa* | JN640679 | 99% | 41 |
| OTU_33 | *Chionobathyscus dewitti* | HQ712909 | 99% | 33 |
| OTU_34 | *Macrourus whitsoni* | JF265124 | 99% | 33 |
| OTU_35 | *Macrourus whitsoni* | JF265125 | 99% | 33 |
| OTU_36 | *Chionobathyscus dewitti* | HQ712909 | 99% | 32 |
| OTU_37 | *Muraenolepis sp.* | HQ713085 | 99% | 32 |
| OTU_38 | *Chionobathyscus dewitti* | HQ712909 | 99% | 30 |
| OTU_39 | *Magnisudis prionosa* | JN640679 | 99% | 30 |
| OTU_40 | *Chionobathyscus dewitti* | HQ712909 | 99% | 30 |
| OTU_41 | *Chionobathyscus dewitti* | HQ712909 | 99% | 28 |
| OTU_42 | *Chionobathyscus dewitti* | HQ712909 | 99% | 26 |
| OTU_43 | *Chionobathyscus dewitti* | HQ712909 | 99% | 26 |
| OTU_44 | *Macrourus whitsoni* | JF265124 | 99% | 26 |
| OTU_45 | *Chionobathyscus dewitti* | HQ712909 | 99% | 25 |
| OTU_46 | *Chionobathyscus dewitti* | HQ712909 | 99% | 25 |
| OTU_47 | *Chionobathyscus dewitti* | HQ712909 | 99% | 23 |
| OTU_48 | *Macrourus whitsoni* | JF265124 | 99% | 23 |
| OTU_49 | *Bathyraja maccaini* | EU119820 | 99% | 22 |
| OTU_50 | *Anotopterus pharao* | EU148072 | 99% | 22 |
| OTU_51 | *Anotopterus pharao* | EU148072 | 99% | 22 |
| OTU_52 | *Macrourus whitsoni* | JF265124 | 99% | 21 |
| OTU_53 | *Chionobathyscus dewitti* | HQ712909 | 99% | 21 |
| OTU_54 | *Bathyraja maccaini* | EU119820 | 99% | 20 |
| OTU_55 | *Chionobathyscus dewitti* | HQ712909 | 99% | 20 |
| OTU_56 | *Chionobathyscus dewitti* | HQ712909 | 99% | 20 |
| OTU_57 | *Magnisudis prionosa* | JN640679 | 99% | 19 |
| OTU_58 | *Macrourus whitsoni* | JF265124 | 99% | 19 |
| OTU_59 | *Chionobathyscus dewitti* | HQ712909 | 99% | 19 |
| OTU_60 | *Chionobathyscus dewitti* | HQ712909 | 99% | 19 |
| OTU_61 | *Chionobathyscus dewitti* | HQ712909 | 99% | 19 |
| OTU_62 | *Chionobathyscus dewitti* | HQ712909 | 100% | 19 |
| OTU_63 | *Bathyraja maccaini* | EU119820 | 99% | 19 |
| OTU_64 | *Lepidonotothen squamifrons* | EU326368 | 99% | 19 |
| OTU_65 | *Chionobathyscus dewitti* | HQ712909 | 99% | 18 |
| OTU_66 | *Chionobathyscus dewitti* | HQ712909 | 99% | 17 |
| OTU_67 | *Macrourus whitsoni* | JF265125 | 99% | 17 |
| OTU_68 | *Chionobathyscus dewitti* | HQ712909 | 99% | 16 |
| OTU_69 | *Chionobathyscus dewitti* | HQ712909 | 99% | 16 |
| OTU_70 | *Chionobathyscus dewitti* | HQ712909 | 99% | 16 |
| OTU_71 | *Chionobathyscus dewitti* | HQ712909 | 99% | 15 |
| OTU_72 | *Chionobathyscus dewitti* | HQ712909 | 99% | 15 |
| OTU_73 | *Muusoctopus (Benthoctopus) levis* | EF016332 | 99% | 15 |
| OTU_74 | *Chionobathyscus dewitti* | HQ712909 | 99% | 15 |
| OTU_75 | *Macrourus whitsoni* | JF265125 | 99% | 15 |
| OTU_76 | *Macrourus whitsoni* | JF265124 | 99% | 15 |
| OTU_77 | *Lepidonotothen squamifrons* | EU326368 | 99% | 15 |
| OTU_78 | *Macrourus whitsoni* | JF265124 | 99% | 14 |
| OTU_79 | *Chionobathyscus dewitti* | HQ712909 | 99% | 14 |
| OTU_80 | *Chionobathyscus dewitti* | HQ712909 | 99% | 14 |
| OTU_81 | *Macrourus whitsoni* | JF265124 | 99% | 14 |
| OTU_82 | *Macrourus whitsoni* | JF265124 | 99% | 14 |
| OTU_83 | *Chionobathyscus dewitti* | HQ712909 | 99% | 14 |
| OTU_84 | *Chionobathyscus dewitti* | HQ712909 | 99% | 14 |
| OTU_85 | *Chionobathyscus dewitti* | HQ712909 | 99% | 13 |
| OTU_86 | *Lepidonotothen squamifrons* | EU326368 | 99% | 13 |
| OTU_87 | *Muraenolepis sp.* | HQ713085 | 99% | 13 |
| OTU_88 | *Chionobathyscus dewitti* | HQ712909 | 99% | 13 |
| OTU_89 | *Chionobathyscus dewitti* | HQ712909 | 99% | 13 |
| OTU_90 | *Chionobathyscus dewitti* | HQ712909 | 99% | 13 |
| OTU_91 | *Macrourus whitsoni* | JF265125 | 99% | 13 |
| OTU_92 | *Chionobathyscus dewitti* | HQ712909 | 99% | 12 |
| OTU_93 | *Macrourus whitsoni* | JF265124 | 99% | 12 |
| OTU_94 | *Chionobathyscus dewitti* | JN640815 | 99% | 12 |
| OTU_95 | *Chionobathyscus dewitti* | HQ712909 | 99% | 12 |
| OTU_96 | *Macrourus whitsoni* | JF265124 | 99% | 12 |
| OTU_97 | *Chionobathyscus dewitti* | HQ712909 | 99% | 12 |
| OTU_98 | *Chionobathyscus dewitti* | JN640815 | 99% | 12 |
| OTU_99 | *Lepidonotothen squamifrons* | EU326368 | 99% | 12 |
| OTU_100 | *Chionobathyscus dewitti* | HQ712909 | 98% | 12 |
| OTU_101 | *Chionobathyscus dewitti* | HQ712909 | 99% | 12 |
| OTU_102 | *Chionobathyscus dewitti* | HQ712909 | 99% | 12 |
| OTU_103 | *Macrourus whitsoni* | JF265124 | 100% | 12 |
| OTU_104 | *Chionobathyscus dewitti* | HQ712909 | 99% | 12 |
| OTU_105 | *Macrourus whitsoni* | JF265125 | 99% | 12 |
| OTU_106 | *Macrourus whitsoni* | JF265125 | 99% | 12 |
| OTU_107 | *Chionobathyscus dewitti* | HQ712909 | 99% | 11 |
| OTU_108 | *Macrourus whitsoni* | JF265124 | 99% | 11 |
| OTU_109 | *Chionobathyscus dewitti* | HQ712909 | 99% | 11 |
| OTU_110 | *Chionobathyscus dewitti* | HQ712909 | 99% | 11 |
| OTU_111 | *Chionobathyscus dewitti* | HQ712909 | 99% | 11 |
| OTU_112 | *Magnisudis prionosa* | JN640679 | 99% | 11 |
| OTU_113 | *Chionobathyscus dewitti* | HQ712909 | 99% | 11 |
| OTU_114 | *Chionobathyscus dewitti* | HQ712909 | 99% | 11 |
| OTU_115 | *Chionobathyscus dewitti* | HQ712909 | 99% | 11 |
| OTU_116 | *Chionobathyscus dewitti* | HQ712909 | 99% | 10 |
| OTU_117 | *Anotopterus pharao* | EU148072 | 99% | 10 |
| OTU_118 | *Chionobathyscus dewitti* | HQ712909 | 99% | 10 |
| OTU_119 | *Chionobathyscus dewitti* | HQ712909 | 99% | 10 |
| OTU_120 | *Chionobathyscus dewitti* | JN640824 | 99% | 10 |
| OTU_121 | *Lepidonotothen squamifrons* | EU326365 | 99% | 10 |
| OTU_122 | *Macrourus whitsoni* | JF265124 | 99% | 10 |
| OTU_123 | *Macrourus whitsoni* | JF265124 | 99% | 10 |
| OTU_124 | *Macrourus whitsoni* | JF265124 | 99% | 10 |
| OTU_125 | *Macrourus whitsoni* | JF265124 | 99% | 10 |
| OTU_126 | *Muusoctopus (Benthoctopus) levis* | EF016332 | 99% | 10 |
| OTU_127 | *Chionobathyscus dewitti* | HQ712909 | 99% | 10 |
| OTU_128 | *Macrourus whitsoni* | JF265124 | 99% | 10 |
| OTU_129 | *Chionobathyscus dewitti* | HQ712909 | 99% | 10 |
| OTU_130 | *Lepidonotothen squamifrons* | EU326365 | 98% | 10 |
