# Supplementary material for: Metabarcoding analysis of the stomach contents of the Antarctic Toothfish (Dissostichus mawsoni) collected in the Antarctic Ocean
Source: PeerJ. 2017 Nov 7;5:e3977. doi: 10.7717/peerj.3977 (PMC5680711; doi:10.7717/peerj.3977)
Supplement: Data S5 [file peerj-05-3977-s005.docx]

| OTU | Species | GenBank | Identity | contigs |
| --- | --- | --- | --- | --- |
| OTU_1 | *Macrourus whitsoni* | JF265125 | 100% | 50362 |
| OTU_2 | *Macrourus whitsoni* | JF265124 | 100% | 8359 |
| OTU_3 | *Chionobathyscus dewitti* | HQ712909 | 99% | 2861 |
| OTU_4 | *Lycenchelys sp.* | JN641010 | 100% | 2709 |
| OTU_5 | *Neopagetopsis ionah* | HQ713088 | 100% | 2020 |
| OTU_6 | *Chionobathyscus dewitti* | JN640824 | 100% | 1794 |
| OTU_7 | *Bathyraja maccaini* | EU119820 | 100% | 1291 |
| OTU_8 | *Psychroteuthis sp. (glacialis)* | AY557544 | 100% | 1135 |
| OTU_9 | *Trematomus lepidorhinus* | HQ713329 | 99% | 549 |
| OTU_10 | *Pogonophryne sp.* | HQ713191 | 99% | 541 |
| OTU_11 | *Trematomus lepidorhinus* | HQ713303 | 100% | 385 |
| OTU_12 | *Pogonophryne scotti* | HQ713186 | 100% | 358 |
| OTU_13 | *Macrourus whitsoni* | JF265125 | 99% | 352 |
| OTU_14 | Unknown | KU558720 | 85% | 349 |
| OTU_15 | *Cirroctopus sp.* | GU073528 | 100% | 267 |
| OTU_16 | *Lycenchelys sp.* | JN641010 | 99% | 250 |
| OTU_17 | *Muraenolepis sp.* | HQ713085 | 100% | 177 |
| OTU_18 | *Macrourus whitsoni* | JF265125 | 99% | 152 |
| OTU_19 | *Macrourus whitsoni* | JF265124 | 99% | 140 |
| OTU_20 | *Cirroctopus sp.* | GU073525 | 99% | 136 |
| OTU_21 | *Macrourus whitsoni* | JF265124 | 99% | 96 |
| OTU_22 | *Cirroctopus sp.* | GU073525 | 99% | 67 |
| OTU_23 | *Benthoctopus sp.* | GU073624 | 100% | 57 |
| OTU_24 | *Macrourus whitsoni* | JF265125 | 99% | 56 |
| OTU_25 | *Macrourus whitsoni* | JF265124 | 99% | 49 |
| OTU_26 | *Chionobathyscus dewitti* | HQ712909 | 99% | 48 |
| OTU_27 | *Macrourus whitsoni* | JF265125 | 99% | 48 |
| OTU_28 | *Macrourus whitsoni* | JF265124 | 99% | 47 |
| OTU_29 | *Macrourus whitsoni* | JF265125 | 99% | 46 |
| OTU_30 | *Macrourus whitsoni* | JF265125 | 99% | 46 |
| OTU_31 | *Pogonophryne sp.* | JN641104 | 99% | 38 |
| OTU_32 | *Chionobathyscus dewitti* | HQ712909 | 99% | 35 |
| OTU_33 | *Macrourus whitsoni* | JF265125 | 99% | 35 |
| OTU_34 | *Macrourus whitsoni* | JF265125 | 99% | 34 |
| OTU_35 | *Macrourus whitsoni* | JF265125 | 99% | 33 |
| OTU_36 | *Macrourus whitsoni* | JF265124 | 99% | 33 |
| OTU_37 | *Macrourus whitsoni* | JF265125 | 99% | 27 |
| OTU_38 | *Pogonophryne sp.* | HQ713191 | 99% | 27 |
| OTU_39 | *Macrourus whitsoni* | JF265125 | 99% | 26 |
| OTU_40 | *Psychroteuthis sp. (glacialis)* | AY557544 | 99% | 25 |
| OTU_41 | *Macrourus whitsoni* | JF265125 | 99% | 25 |
| OTU_42 | *Macrourus whitsoni* | JF265125 | 99% | 24 |
| OTU_43 | *Macrourus whitsoni* | JF265125 | 99% | 21 |
| OTU_44 | *Pogonophryne scotti* | JN641123 | 100% | 21 |
| OTU_45 | *Macrourus whitsoni* | JF265124 | 99% | 19 |
| OTU_46 | *Chionobathyscus dewitti* | JN640815 | 99% | 19 |
| OTU_47 | *Macrourus whitsoni* | JF265124 | 99% | 19 |
| OTU_48 | *Macrourus whitsoni* | JF265125 | 99% | 18 |
| OTU_49 | *Macrourus whitsoni* | JF265125 | 99% | 18 |
| OTU_50 | *Macrourus whitsoni* | JF265125 | 99% | 18 |
| OTU_51 | *Macrourus whitsoni* | JF265124 | 99% | 18 |
| OTU_52 | *Chionobathyscus dewitti* | JN640824 | 99% | 18 |
| OTU_53 | *Macrourus whitsoni* | JF265125 | 99% | 17 |
| OTU_54 | *Macrourus whitsoni* | JF265124 | 99% | 17 |
| OTU_55 | *Macrourus whitsoni* | JF265124 | 99% | 17 |
| OTU_56 | *Graneledone antarctica* | AF377973 | 100% | 16 |
| OTU_57 | *Trematomus lepidorhinus* | HQ713303 | 99% | 16 |
| OTU_58 | *Macrourus whitsoni* | JF265125 | 99% | 16 |
| OTU_59 | *Macrourus whitsoni* | JF265125 | 99% | 16 |
| OTU_60 | *Macrourus whitsoni* | JF265125 | 99% | 15 |
| OTU_61 | *Macrourus whitsoni* | JF265124 | 99% | 15 |
| OTU_62 | *Macrourus whitsoni* | JF265125 | 99% | 15 |
| OTU_63 | *Macrourus whitsoni* | JF265125 | 99% | 15 |
| OTU_64 | *Macrourus whitsoni* | JF265125 | 99% | 15 |
| OTU_65 | *Macrourus whitsoni* | JF265124 | 99% | 14 |
| OTU_66 | *Macrourus whitsoni* | JF265124 | 99% | 14 |
| OTU_67 | *Macrourus whitsoni* | JF265125 | 99% | 14 |
| OTU_68 | *Macrourus whitsoni* | JF265124 | 99% | 14 |
| OTU_69 | *Chionobathyscus dewitti* | JN640815 | 99% | 13 |
| OTU_70 | *Macrourus whitsoni* | JF265124 | 99% | 13 |
| OTU_71 | *Macrourus whitsoni* | JF265124 | 99% | 13 |
| OTU_72 | *Macrourus whitsoni* | JF265125 | 99% | 13 |
| OTU_73 | *Macrourus whitsoni* | JF265124 | 99% | 13 |
| OTU_74 | *Macrourus whitsoni* | JF265124 | 99% | 13 |
| OTU_75 | *Macrourus whitsoni* | JF265125 | 99% | 13 |
| OTU_76 | *Macrourus whitsoni* | JF265125 | 99% | 13 |
| OTU_77 | *Macrourus whitsoni* | JF265125 | 99% | 13 |
| OTU_78 | *Macrourus whitsoni* | JF265125 | 99% | 12 |
| OTU_79 | *Macrourus whitsoni* | JF265125 | 99% | 12 |
| OTU_80 | *Macrourus whitsoni* | JF265124 | 99% | 12 |
| OTU_81 | *Macrourus whitsoni* | JF265124 | 99% | 12 |
| OTU_82 | *Lycenchelys sp.* | JN641010 | 99% | 12 |
| OTU_83 | *Macrourus whitsoni* | JF265124 | 99% | 12 |
| OTU_84 | *Macrourus whitsoni* | JF265124 | 99% | 12 |
| OTU_85 | *Macrourus whitsoni* | JF265124 | 99% | 12 |
| OTU_86 | *Macrourus whitsoni* | JF265125 | 99% | 11 |
| OTU_87 | *Macrourus whitsoni* | JF265125 | 99% | 11 |
| OTU_88 | *Macrourus whitsoni* | JF265125 | 99% | 11 |
| OTU_89 | *Macrourus whitsoni* | JF265125 | 99% | 11 |
| OTU_90 | *Neopagetopsis ionah* | HQ713088 | 99% | 10 |
| OTU_91 | *Macrourus whitsoni* | JF265124 | 99% | 10 |
| OTU_92 | *Chionobathyscus dewitti* | JN640824 | 99% | 10 |
| OTU_93 | *Macrourus whitsoni* | JF265125 | 99% | 10 |
| OTU_94 | *Macrourus whitsoni* | JF265125 | 99% | 10 |
| OTU_95 | *Macrourus whitsoni* | JF265125 | 99% | 10 |
| OTU_96 | *Macrourus whitsoni* | JF265124 | 99% | 10 |
| OTU_97 | *Macrourus whitsoni* | JF265125 | 99% | 10 |
| OTU_98 | *Macrourus whitsoni* | JF265124 | 99% | 10 |
| OTU_99 | *Macrourus whitsoni* | JF265124 | 99% | 10 |
| OTU_100 | *Macrourus whitsoni* | JF265125 | 99% | 10 |
| OTU_101 | *Macrourus whitsoni* | JF265125 | 99% | 10 |
| OTU_102 | *Macrourus whitsoni* | JF265125 | 99% | 10 |
| OTU_103 | *Macrourus whitsoni* | JF265124 | 99% | 10 |
| OTU_104 | *Macrourus whitsoni* | JF265125 | 99% | 10 |
| OTU_105 | *Macrourus whitsoni* | JF265124 | 99% | 10 |
